# Pulsed Electric Fields for Emerging Single‐Cell Bioprocessing in Food Applications: Electropermeabilization Mechanisms and Design Principles

**DOI:** 10.1111/1541-4337.70411

**Published:** 2026-02-16

**Authors:** Byron Perez, Julia Baumgartner, Daniel Macken, Iris Haberkorn, Alexander Mathys

**Affiliations:** ^1^ Sustainable Food Processing Laboratory, Institute of Food Nutrition and Health ETH Zurich Zurich Switzerland; ^2^ Singapore‐ETH Centre Singapore Singapore

## Abstract

This review evaluates pulsed electric fields (PEF) as an emerging platform for single‐cell bioprocessing in food applications. Connections are drawn between key mechanisms in electropermeabilization and applications, and a practical PEF process design framework is provided. Electropermeabilization is governed by electrical parameters, physicochemical conditions, cellular traits, and system‐level variables such as chamber geometry and residence time. Furthermore, cell densities and organism choice critically influence processing outcomes. Linking mechanistic understanding and practical applications, this holistic approach aims to increase reproducibility and scalability in PEF‐based single‐cell bioprocessing in the food sector.

## 1 Introduction

Single‐cell organisms are being increasingly investigated for their capacity to produce nutrients, bioactive molecules, and high‐value ingredients (Graham and Ledesma‐Amaro 2023). Their rapid growth rates, minimal arable land requirements, and diverse metabolic capabilities contribute to more resilient and low‐emission production models (Y. Li et al. 2024). For food and feed applications, efficient use of this biomass depends on controlled mass transfer, which governs substrate uptake and the release or recovery of intracellular products (Martínez et al. 2020; Timira et al. 2022).

High‐voltage pulsed electric fields (PEF) is a relatively low‐temperature technology that has been extensively studied for food preservation (Barbosa‐Cánovas et al. 1999). In industrial food processing, PEF is also applied to increase mass transfer in plant and animal tissues by membrane electropermeabilization, including applications in potatoes, fruits, vegetables, and meats (Huang et al. 2025; Peng et al. 2025). In single‐cell bioprocessing, PEF can offer additional functionality by enabling reversible or irreversible permeabilization through appropriate selection of process parameters and system design (Bodénès et al. 2019; Vaessen et al. 2019). Moreover, since PEF often limits bulk heating and does not require chemical agents, it can support selective extraction and delivery while preserving sensitive biomolecules (Grimi et al. 2014; Suarez Garcia et al. 2018).

Despite this potential, PEF outcomes remain difficult to transfer across studies and systems. For example, controlled permeabilization of microalgae has been reported to reduce debris formation and facilitate protein recovery by microfiltration (Perez, Li, et al. 2025; Safi et al. 2017), although performance at high cell concentrations still requires further optimization (Krust et al. 2022; Perez, Azzari, et al. 2025; Pucihar et al. 2007). For nutrient enrichment, PEF has been used to load yeast cells with vitamins and minerals, demonstrating its potential for developing nutrient delivery systems in other organisms (Góral et al. 2019; Nowosad et al. 2021, 2023). PEF has also been studied for extraction intensification, yet outcomes remain case‐dependent, with mixed yields and limited transferability across organisms and solvent systems (Antezana et al. 2013; Gorte et al. 2020; Lai et al. 2014; Sommer et al. 2021). This limited transferability reflects the coupled effects of electrical parameters, biological traits, medium properties, and engineering design.

Biological characteristics strongly influence PEF susceptibility and the resulting mass transfer. Cell size and anisotropy, membrane composition, and the presence of a cell wall can shape electric field interactions, charging dynamics, and transport pathways (Breton and Mir 2018; EL‐Hag and Jayaram 2008; Jacobs et al. 2023; Ye et al. 2024). The cell wall, in particular, regulates the release of intracellular compounds and can be modified by the treatment, either directly or through secondary effects (Bensalem et al. 2020; Buchmann et al. 2019; Ganeva et al. 2014; Stirke et al. 2019). In parallel, physicochemical conditions such as osmolarity, pH, and conductivity shape permeabilization and resealing, while also influencing Joule heating and its effects on cell survival and product stability (Delso et al. 2022; Gančytė et al. 2023; Ivorra et al. 2010; Y. Li et al. 2015).

Engineering aspects, including chamber design, fluid flow, and electrode configuration, determine the spatial and temporal delivery of the electric field (Raso et al. 2016). Homogeneous conditions are needed to achieve reproducible permeabilization by delivering a defined number of pulses at a defined field strength (García‐Sánchez et al. 2019; Muratori et al. 2017; Raso et al. 2016). Nevertheless, chamber geometry and electrode layout create gradients in field strength and residence time, leading to uneven treatment (Buchmann et al. 2018; Zand et al. 2021). Moreover, transferability and reproducibility are hindered by the nonstandard reporting of key parameters, which limits cross‐study comparisons and process reproducibility (Raso et al. 2016).

This review examines reversible and irreversible PEF‐based electropermeabilization in single‐cell bioprocessing, integrating biological, physicochemical, and engineering perspectives. It highlights how mechanisms and design factors influence mass transfer outcomes and outlines strategies for optimizing parameters, chamber design, and standardized reporting. Finally, it proposes transferable design principles by linking mechanistic and technical advances with system design and process conditions, aiming to support reproducibility, scalability, and energy efficiency in PEF‐based single‐cell bioprocessing for food applications.

## 2 Literature Selection Methodology

The literature for this structured narrative review was identified in Scopus. The final search was conducted in December 2025 and limited to English‐language journal articles published from 2010 to 2025. For single‐cell applications, the Scopus query was: TITLE ABS KEY (pulsed electric field OR PEF OR electroporation OR electropermeabilization) AND TITLE ABS KEY (yeast OR yeasts OR microalga OR microalgae OR bacteria OR bacterial) AND NOT TITLE ABS KEY (inactivation OR preservation). Records were screened by title, abstract, and methodology. Studies were included when they were considered relevant examples of pulsed electric fields‐based extraction or mass transfer enhancement in microalgae, yeasts, or bacteria and reported at least pulse width and electric field strength. Electrical, engineering, and biological parameters were extracted when available, and missing information was recorded as not reported.

For electropermeabilization mechanisms, additional Scopus searches combined the same pulsed electric field terms with topic‐specific keywords covering electrical settings, physicochemical conditions, and cellular responses, including conductivity, osmolarity, temperature, cell wall, cell membrane, and so forth. Because mechanistic evidence in microbial single‐cell systems remains limited, studies on animal cells, protoplasts, and model membranes were included when they supported the interpretation and could be linked to single‐cell observations. Key references published before 2010 were also included when directly relevant. For engineering considerations, searches focused on continuous‐flow processing, including residence time, flow effects, treatment‐chamber design, and reporting of pulse and field parameters, with emphasis on food‐processing studies.

## 3 Mechanisms Underlying Pulsed Electric Field‐Based Electropermeabilization

In PEF treatments, short electric pulses (≲ 2 ms) with high electric field strengths (often 0.1 to 100 kV cm^−1^) are applied to a substrate placed between electrodes. When this substrate consists of a cell suspension, the electric field induces charge accumulation across the cells’ membranes. Two connected processes contribute to membrane permeabilization by PEF, both falling under the broader term electropermeabilization as reviewed by Kotnik et al. (2019). The first is electroporation, which involves the formation of temporary pores in the lipid bilayer, either hydrophilic or hydrophobic. These pores remain open while the electric field is applied and close shortly after, as their stability depends directly on external energy input (Mahnič‐Kalamiza and Miklavčič 2022). Although the terms PEF treatment and electroporation are often used interchangeably (Kotnik et al. 2019), electroporation refers explicitly to this immediate, reversible event. A second, more complex process results in the creation of long‐lasting permeable sites caused by additional effects of the electric field on the membrane and surrounding environment (Breton and Mir 2018; Graybill and Davalos 2020; Kotnik et al. 2019; Vernier et al. 2009). These persistent sites usually form at locations initially affected by electroporation (Silkunas et al. 2024), and both phenomena, electroporation and additional effects, cannot be distinguished from each other except under specific experimental conditions with artificial bilayers (Breton and Mir 2018; Delemotte and Tarek 2012; Thompson et al. 2014). Together, these reversible and prolonged changes in membrane permeability define the overall process of electropermeabilization.

### 3.1 The Phenomenon of Electroporation During Electropermeabilization

The primary physical driver of electroporation is the electric field strength *E* [V m^−1^] (Kotnik et al. 2012), which is often reported as the voltage‐to‐distance ratio. In ideal parallel plate treatment chambers (most used research set‐ups), the nominal field can be defined as the applied voltage *U(t)* [V] divided by the electrode gap *L* [m] (Equation 1):

(1) $$E\left(t\right)=\frac{U\left(t\right)}{L}\;$$

This definition applies to both square and exponentially decaying pulses, where *U(t)* may represent a constant voltage (square pulse) or a time‐dependent decay function. In other chamber configurations, the local field is not uniform across the treatment zone. Therefore, *E* should be treated as a representative value and, when needed, verified by measurement or simulation (Knappert et al. 2020; Schottroff et al. 2020; Raso et al. 2016).

*E* induces a transmembrane potential *ΔΨ_ind_* [V] that adds to the cell's resting transmembrane potential *ΔΨ_rest_* [V] to give the total transmembrane potential *ΔΨ_total_* [V] (Equation 2):

(2) $$\Delta \Psi_{\mathrm{total}}=\Delta \Psi_{\mathrm{rest}}+\Delta \Psi_{\mathrm{ind}}$$

The value of *ΔΨ_rest_*, which depends on the species‐specific equilibrium with their environment, is typically negative in most non‐extremophile cells (≈ –200 mV) and small relative to *ΔΨ_ind_*. Therefore, it is generally assumed that *ΔΨ_ind_* ≈ *ΔΨ_total_*. Electroporation occurs when *ΔΨ_total_* exceeds a critical threshold *ΔΨ_crit_* [V]. The critical threshold can vary widely depending on the membrane fluidity, which reflects the physical flexibility and lipid mobility within the bilayer, but is often around 1 V for many cell types in their standard environment (Kotnik et al. 2012).

**FIGURE 1:**
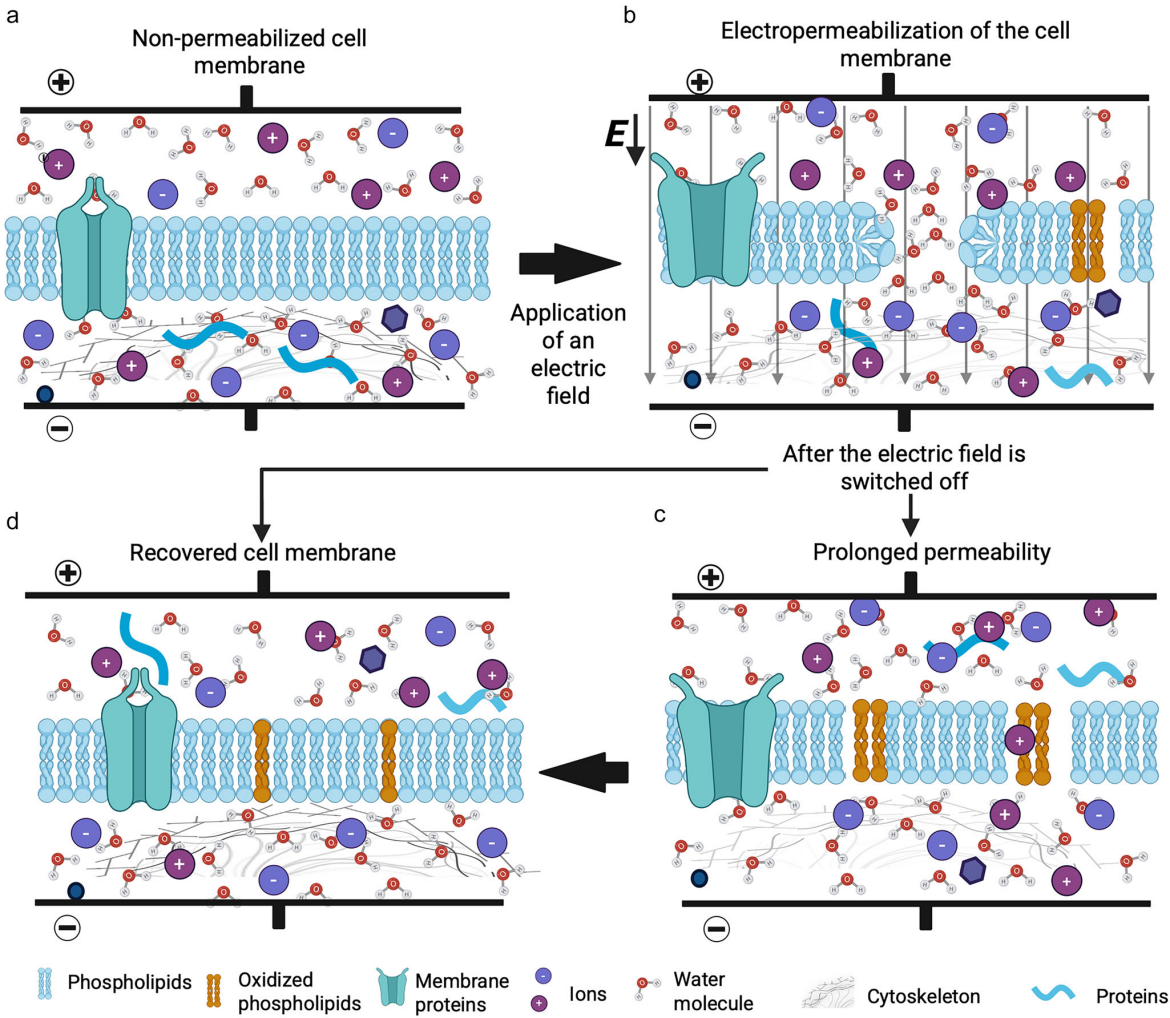
Mechanisms of electropermeabilization. In the absence of an electric field, the cell membrane remains intact, preventing uncontrolled mass transfer a). When the applied electric field exceeds the transmembrane voltage threshold, electropermeabilization occurs, a combination of phospholipid rearrangement that leads to hydrophilic pore formation (electroporation), accompanied by other cellular effects, including lipid oxidation, protein denaturation, and cytoskeletal damage b). While transient hydrophilic pores close within microseconds after the removal of the electric field, structural damage can cause prolonged permeability c). Under reversible conditions, membrane integrity is restored d); however, excessive damage may result in irreversible disruption and cell death.

When *ΔΨ_total_* is exceeded, transient hydrophobic electropores form as aligned water molecules penetrate the membrane core (Ye et al. 2024). This intermediate state is energetically unfavorable due to the membrane core's intrinsic hydrophobicity (Delemotte and Tarek 2012). Subsequent phospholipid reorientation exposes polar head groups (Figure 1), creating hydrophilic pores that stabilize the opening and permit the passage of water, ions, and small molecules (Kotnik et al. 2019; Mahnič‐Kalamiza and Miklavčič 2022). The time course of *ΔΨ_ind_* for a square pulse is described by the first‐order Schwan equation (Kotnik et al. 2019; Pauly and Schwan 1959; Equation 3):

(3) $$\Delta \Psi_{\mathrm{ind}}=f_{r}\;\cdot E\cdot r\cdot \cos\theta \cdot \left(1-\exp\left(\frac{-t}{\tau_{m}}\right)\right)$$

where *f_r_* is the cell shape factor, *r* [m] is the cell radius, *θ* is the angle between *E* and the membrane surface, and *τ_m_* [s] is the membrane charging time constant. If the pulse duration is shorter than *τ_m_*, the membrane cannot reach the *ΔΨ_crit_* for electroporation, and permeabilization may not occur, although additional membrane damage due to *E* effects on other structures may still occur. *τ_m_* can be estimated from the Maxwell–Wagner model (Equation 4):

(4) $$\tau_{m}=\frac{r\cdot \varepsilon_{m}}{2d_{m}\frac{\sigma_{i}\cdot \sigma_{e}}{\sigma_{i}+2\sigma_{e}}+r\cdot \sigma_{m}}\;$$

Here, *ε_m_* [F m^−1^] is the permittivity of the membrane and *σ_i_*, *σ_m_*, *σ_e_* [S m^−1^] are the conductivity of the cytoplasm, membrane, and surrounding medium, and *d_m_* [m] is the membrane thickness. When sufficiently long pulses (> several µs) are applied (Kotnik et al. 2012, 2019) and the suspension conductivity exceeds 0.01 mS cm^−1^ (Ivorra et al. 2010), *τ_m_* becomes negligible relative to the exposure time, and *ΔΨ_ind_* (Equation 3) simplifies to Equation (5):

(5) $$\Delta \Psi_{\mathrm{ind}}=f_{r}\cdot E\cdot r\cdot \cos\theta$$

The distribution and magnitude of *ΔΨ_ind_* are influenced by cell geometry and orientation relative to *E* (Valic et al. 2003). Smaller cells will require a higher *E* to reach the same *ΔΨ_ind_* (EL‐Hag and Jayaram 2008). Moreover, depending on cell shape, *f_r_* differs due to charge distribution, with spherical cells having *f_r_* = 3/2 (Kotnik 2016). Not only is the distribution of charge affected by the shape, but the area where *E* will produce higher *ΔΨ_ind_* in spherical cells reaches a maximal effect at the poles (*θ* = 0°) and minimal values at the equator, depending on the contact angle with *E* (Pavlin et al. 2002). For cylindrical cells aligned perpendicular to *E*, such as in the case of *Arthrospira platensis* aligned with the flow due to shear forces (Buchmann et al. 2018), *f_r_* can be approximated as *f_r_* = 2 (Kotnik 2016). These cells will also present a larger area exposed to *E*, potentially increasing their susceptibility to permeabilization.

Hydrophilic electropores are short‐lived because an open pore is energetically unfavorable once the electric field is removed. In lipid membranes, resealing can occur within milliseconds (Valic et al. 2003). Under ideal conditions, the membrane therefore returns to a low permeability state after the pulse (Riske and Dimova 2005; Tieleman 2004). However, permeable regions can also reflect co‐localized changes in membrane lipids and proteins, and in the underlying cytoskeleton, which are challenging to separate experimentally from pore formation (Silkunas et al. 2024; Kotnik et al. 2019). If these permeable sites do not recover, the outcome is often termed irreversible electroporation, but a more accurate term is irreversible electropermeabilization, given the additional effects caused by the electric field. When the goal is to describe inactivation, it is also important to consider that irreversible electropermeabilization is not the only mechanism leading to cell death, but it involves distinct biological responses and cell death pathways, including necrosis, apoptosis, and pyroptosis (Batista Napotnik et al. 2021). Cell death mechanisms are outside the scope of this review, but Section 3.2 discusses permeability effects beyond pore formation that can sustain transport after pulsing.

### 3.2 Cellular Effects Beyond Pore Formation Leading to Electropermeabilization

The sustained increase in membrane permeability observed minutes to hours after PEF treatment involves additional, complex structural and functional changes, which have been reviewed in detail by Kotnik et al. (2019). These effects, which are not yet fully understood by current models, include lipid peroxidation, membrane protein damage, and cytoskeletal disruption (Breton and Mir 2018; Graybill and Davalos 2020; Vernier et al. 2009).

At the membrane level, lipid peroxidation plays a crucial role in long‐lasting changes in membrane permeability, caused by the oxidative degradation of polyunsaturated fatty acids (Breton and Mir 2018). Reactive oxygen species initiate these oxidative processes, which are generated both intracellularly, such as from mitochondria or oxidase enzymes, and via electrogenerated radicals formed at the electrode–electrolyte interface (e.g., superoxide, H_2_O_2_) during PEF treatment (Vernier et al. 2009). Membrane protein damage at high *E* (often 0.1 to 100 kV cm^−1^) also contributes to the formation of complex permeable sites, leading to longer‐lasting permeability changes (Blažič et al. 2025). PEF can also disrupt the cytoskeleton, which prolongs membrane permeability (Graybill and Davalos 2020). During PEF, electrophoretic forces disrupt actin filaments, lowering the energy barrier to pore formation and increasing permeability (Muralidharan et al. 2021; Perrier et al. 2019).

In summary, membrane permeabilization induced by PEF arises from electropermeabilization, which includes rapid pore formation during the pulse, depending on the field strength, and additional disruptive effects on membrane lipids, surface proteins, and the cytoskeleton that occur during and after the pulse. These secondary effects can prolong permeability through structural and biochemical changes (Figure 1). *E* governs the entire process, from the development of the transmembrane potential to the secondary impacts on membrane components and the cytoskeleton. The field can be delivered in different ways depending on the application, as discussed in the next section.

## 4 Applications of Pulsed Electric Fields in Single‐Cell Bioprocessing

Optimizing the parameters that define electric field delivery, including pulse duration, pulse number, and waveform, has enabled PEF to be applied across diverse single‐cell systems and processing goals. Early studies focused on microbial inactivation, where PEF can reduce thermal load compared with conventional heat treatments and help retain product quality (Barbosa‐Cánovas et al. 1999; Ghoshal 2023). Competitive inactivation has been demonstrated in model bacteria such as *Escherichia coli*, *Bacillus megaterium*, and *Listeria innocua*, with strong dependence on treatment temperature and the rate of temperature rise during processing (Toepfl et al. 2007). Key limitations and future PEF‐inactivation research focus include the lower sensitivity of bacterial spores, the presence of survivors, and matrix‐specific constraints that affect field distribution, heating, and cellular stress responses (Timmermans et al. 2014; Nabilah et al. 2022). In contrast, this review emphasizes emerging food bioprocessing applications in which PEF induced permeabilization enhances mass transfer to enable extraction, recovery, and intracellular delivery.

**FIGURE 2:**
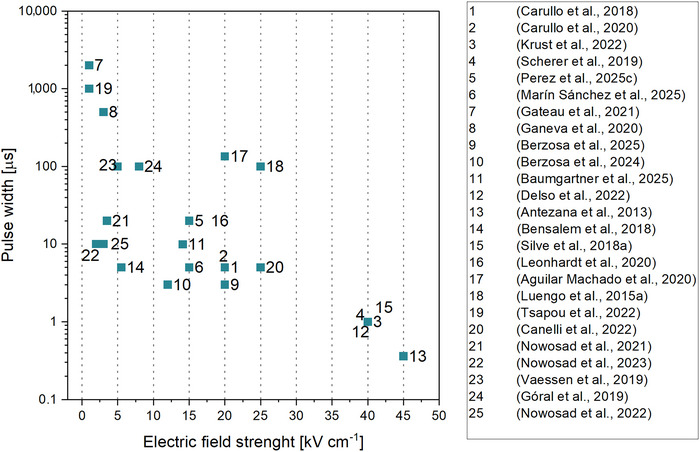
Reported combinations of electric field strength and pulse width used in pulsed electric field (PEF) treatments for microalgae, yeast, and bacteria in single‐cell bioprocessing. Each numbered data point corresponds to a study listed in the legend, covering applications including reversible permeabilization, protein extraction, lipid recovery, pigment release, and nutrient bioenrichment.

The parameter space reported for single‐cell bioprocessing spans a wide range of biological diversity and electrical conditions (Table 1). Electric field strengths range from 1 kV cm^−1^ in *Haematococcus pluvialis* treated for reversible permeabilization (Gateau et al. 2021) to 45 kV cm^−1^ in *Ankistrodesmus falcatus* for enhanced lipid extraction (Antezana et al. 2013). Specific energy inputs similarly vary from about 1.5 kJ kg_sus_^−1^ in lipid‐related processing of *Chlamydomonas reinhardtii* (Bensalem et al. 2018) to around 150 kJ kg_sus_^−1^ in several extraction studies targeting proteins or lipids (Scherer et al. 2019; Silve, Kian, et al. 2018; Delso et al. 2022). Across studies, pulse duration and field strength typically trade off. Short pulses are combined with higher fields, whereas longer pulses are applied at lower fields. Many food‐related applications use microsecond pulses with fields of about 15–25 kV cm^−1^ (Figure 2), but the effective window remains application‐ and organism‐specific.

**TABLE 1:** Pulsed electric fields applications in single‐cell bioprocessing.

	Application	Organism	Electric conditions	Outcome		Reference
	Extraction			Value	Normalization basis	
**1**	Protein and carbohydrates	Microalgae *Chlorella vulgaris* (12 g L^−1^)	*E*: 20 kV cm^−1^; *t_p_*: 5 µs; *W_s_*: 100 kJ kg_sus_^−1^; *n*: n.r.; PS: square monopolar; CH: continuous co‐linear	Proteins: 5.2% (w/w); carbohydrates: 35.8% (w/w)	Relative to total extractable fraction defined by HPH	Carullo et al. (2018)
**2**	Water‐soluble proteins, C‐phycocyanin, and carbohydrates	Cyanobacteria *Arthrospira platensis* (20 g L^−1^)	*E*: 20 kV cm^−1^; *t_p_*: 5 µs; *W_s_*: 100 kJ kg_sus_^−1^; *n*: n.r.; PS: square monopolar; CH: continuous co‐linear	Water soluble proteins: 25.4% (w/w); carbohydrates: 64.1% (w/w); C phycocyanin: 37.4% (w/w)	Relative to total extractable fraction defined by HPH	Carullo et al. (2020)
**3**	Protein	Microalgae *Chlorella vulgaris* (7‐10 g L^−1^)	*E*: 40 kV cm^−1^; *t_p_*: 1 µs; *n*: 16; *W_s_*: 9.4 kJ kg_sus_^− 1^ PS: square monopolar; CH: continuous parallel plate	Protein: 22% (w/w) after 24 h incubation	Biomass dry weight basis	Krust et al. (2022)
**4**	Protein	Microalgae *Chlorella vulgaris (7‐10 *g L^−1^)	*E*: 40 kV cm^−1^; *t_p_*: 1 µs; *n*: 47 *W_s_*: 150 kJ kg_sus_^−1^ PS: square monopolar; CH: continuous parallel plate	Protein: 22% (w/w) after 24 h incubation	Biomass dry weight basis	Scherer et al. (2019)
**5**	Reversible permeabilization for protein	Microalgae *Auxenochlorella protothecoides *(9.3 ± 0.7 g L^−1^)	*E*: 15 kV cm^−1^; *t_p_*: 20 µs; *n*: 5; *W_s_*: 15.42 kJ kg_sus_^−1^; PS: square monopolar; CH: batch parallel plate	Protein: 1.1% (w/w) after 1 h incubation	Biomass dry weight basis	Perez, Weber, et al. (2025)
**6**	Protein	Yeast *Saccharomyces cerevisiae* (22.17 ± 0.06 g L^−1^)	*E*: 15 kV cm^−1^; *t_p_*: 5 µs; *n*: 19; *W_s_*: 43.3 kJ kg_sus_^−1^; PS: square monopolar CH: continuous parallel plate	Total proteins: ∼80% (w/w) after 24 h incubation	Relative to total extractable protein defined by bead milling	Marín‐Sánchez et al. (2025)
**7**	Reversible permeabilization for protein	*Microalgae Haematococcus pluvialis* (10^5^ cells mL^−1^)	*E*: 1 kV cm^−1^; *n*: 5 or 9 *t_p_*: 2 ms; *W_s_*: 51.5 kJ kg_sus_^−1^ PS: bipolar square; CH: batch parallel plate	Total proteins: 46% (w/w)	Relative to total extractable protein defined by ultrasonication	Gateau et al. (2021)
**8**	Protein	Yeast *Saccharomyces cerevisiae* (66 g L^−1^)	*E*: 3 kV cm^− 1^; *t_p_*: 0.5 ms; *n*: 19; *W_s_*: 120 kJ kg^−1^ PS: square monopolar; CH: continuous flow parallel plate	Protein: 87.8% ± 3% (w/w)	Relative to total extractable protein defined by bead milling	Ganeva et al. (2020)
**9**	Autolysis products	Yeast *Saccharomyces cerevisiae* (29 g L^−1^)	*E*: 20 kV cm^− 1^; *t_p_*: 3 µs; *n*: 50; *W_s_*: 150 kJ kg^−1^ PS: square monopolar; CH: continuous flow parallel plate	Glutathione: 70 to 75% (w/w); proteins: 25 to 40% (w/w); protein after 24 h: ∼50% (w/w)	Relative to total extractable fraction defined by bead milling	Berzosa et al. (2025)
**10**	Glutathione	Yeast *Saccharomyces cerevisiae* (22.41 ± 3.47 g L^−1^)	*E*: 12 kV cm^−1^; *t_p_*: 3 µs; *n*: 50 (total treatment time 150 µs); *W_s_*: 55.3 ± 5.1 kJ kg_sus_^−1^; PS: square monopolar; CH: continuous flow parallel plate	Glutathione: 78% (w/w) after 24 h incubation	Relative to total extractable glutathione defined by bead milling	Berzosa et al. (2024)
**11**	Lipid droplets	Microalgae *Chlamydomonas reinhardtii* UVM4 (cell wall‐deficient) (6 g L^−1^)	*E*: 14.1 ± 0.3 kV cm^−1^; *t_p_*: 9.9 ± 0.1 µs; *n*: 9; *W_s_*: 29.7 kJ kg_sus_^−1^ PS: monopolar square; CH: continuous flow parallel plate	Lipids: 42.1% ± 6.8% (w/w)	Relative to total lipid content	Baumgartner et al. (2025)
**12**	Lipid^a^	Microalgae *Auxenochlorella protothecoides* (80 g L^−1^)	*E*: 40 kV cm^−1^; *t_p_*: 1 µs; *n*: 8 *W_s_*: 53.6 kJ kg_sus_^−1^ PS: monopolar square; CH: batch parallel plate	Lipids: 25.8% (w/w) after 6 h incubation	Biomass dry weight basis	Delso et al. (2022)
**13**	Lipid^a^	Microalgae *Ankistrodesmus falcatus* (0.35 g L^−1^)	*E*: 45 kV cm^−1^; *t_p_*: 360 ns; *n*: 7200; *W_s_*: 42 kJ kg_sus_^−1^ PS: square monopolar; CH: continuous flow parallel plate	Lipid extraction: 130% increase	Relative change vs. untreated control	Antezana et al. (2013)
**14**	Lipid^a^	Microalgae *Chlamydomonas reinhardtii* (1 g L^−1^)	*E*: 5.5 kV cm^−1^; *t_p_*: 5 µs; *n*: 10 *W_s_*: 1.51 kJ kg_sus_^−1^ PS: square monopolar; CH: batch parallel plate	Lipids: 88% (w/w) with PEF plus mechanical disruption; 81% untreated	Relative to total lipid content	Bensalem et al. (2018)
**15**	Lipid^a^	Microalgae *Auxenochlorella protothecoides*	*E*: 40 kV cm^−1^; *t_p_*: 1 µs; *W_s_*: 150 kJ kg_sus_^−1^ PS: square monopolar; CH: continuous flow parallel plate	Lipids: 97% (w/w) after 20 h extraction; 10% untreated	Relative to total lipid content	Silve, Papachristou et al. (2018)
**16**	Lipid^a^	Microalgae *Chlorella sorokiniana* (30 g L^−1^)	*E*: 15 kV cm^− 1^; *W_s_*: 100 kJ kg_sus_^−1^ *t_p_*: 20 µs PS: square monopolar; CH: continuous flow co‐linear	No significant increase		Leonhardt et al. (2020)
**17**	Astaxanthin^a^	Yeast *Xanthophyllomyces dendrorhous*	*E*: 20 kV cm^− 1^; *t_p_*: 135 µs; *f*: 0.5 Hz; *W_s_*: 54 kJ kg^−1^ PS: square monopolar; CH: batch parallel plate	Astaxanthin: 2.41 ± 0.12 mg g^−1^	Biomass dry weight basis	Aguilar‐Machado et al. (2020)
**18**	Lutein	Microalgae *Chlorella vulgaris* (2.5 g L^−1^)	*E*: 25 kV cm^− 1^; *t_p_*: 100 µs; *n*: 10; *W_s_*: 50 kJ kg^−1^ PS: square monopolar; CH: batch parallel plate	Lutein: 753 µg g^−1^	Biomass dry weight basis	Luengo, Martínez, Bordetas, et al. (2015)
**19**	Polyols and propanediols	Yeast *Torulaspora delbrueckii*	*E*: 1 kV cm^−1^ *; t_p_*: 1 ms; *f*: 1 Hz; treatment time: 30 min; PS: square monopolar; CH: continuous flow through, coaxial	1,2 propanediol diacetate: extraction increases between 12% and 191% at pH 7.1	Relative change vs. control	Tsapou et al. (2022)
	Other applications					
**20**	Lipid bioaccessibility	Microalgae *Chlorella vulgaris* (20 g L^−1^)	*E*: 20–30 kV cm^−1^; *t_p_*: 5 µs; *n*: 10; *W_s_*: 31.8^−1^49 kJ kg_sus_^− 1^ PS: square monopolar; CH: batch parallel plate	Lipid bioaccessibility: 30% to 50‐60% (w/w)	Bioaccessibility fraction of total lipids	Canelli et al. (2022)
**21**	Mineral enrichment	Yeast *Saccharomyces cerevisiae* (100 g L^−1^)	*E*: 3.5 kV cm^−1^; *t_p_*: 20 µs; *n*: 8; *W_s_*: 36.8 kJ kg_sus_^−1^ PS: square monopolar; CH: batch parallel plate	Iron accumulation: about 55% increase	Relative change vs. untreated control (intracellular iron content)	Nowosad et al. (2021)
**22**	Mineral and vitamin enrichment in yeast biomass	Yeast *Saccharomyces cerevisiae*	*E*: 3 kV cm^−1^; *t_p_*: 10 µs; *n*: 1200; *f*: 1 Hz; PS: square monopolar; CH: batch parallel plate	Iron accumulation: about 148% increase and vitamin B12 accumulation 102%	Relative change vs. untreated control	Nowosad et al. (2023)
**23**	Reversible permeabilization	*Bacteria* *Lactobacillus plantarum* WCFS1 (0.4 g L^−1^)	*E*: 5 kV cm^− 1^; *t_p_*: 100 µs; *n*: 10; *W_s_*: 10 kJ kg_sus_^−1^ PS: square monopolar; CH: batch parallel plate	Permeabilized cells: about 90%	Percent of cells	Vaessen et al. (2019)
**24**	Mineral enrichment	Bacteria *Lactobacillus rhamnosus* (10 ^8^ cells mL^−1^)	*E*: 8 kV cm^− 1^; *t_p_*: 100 µs; *n*: 15; *W_s_*: 96 kJ kg_sus_^−1^ PS: square monopolar; CH: batch parallel plate	Zinc content: up to 73% increase; Permeabilized cells: about 90%	Zinc: relative change vs. untreated control, intracellular zinc content. Percent of total cells	Góral et al. (2019)
**25**	Vitamin enrichment	Yeast *Saccharomyces cerevisiae*	*E*: 1.96 kV cm^−1^; *t_p_*: 10 µs; *n*: 1200*; f*: 1 Hz; PS: unipolar square; CH: batch parallel plate (four electrodes)	Vitamin C: ∼1.3 mg g^−1^ (≈1.7× higher than control)	Biomass dry weight basis	Nowosad et al. (2022)

*Note*: The processing parameters are the electric field *E* [kV cm^−1^] and calculated energy input *Ws* [kJ kg_sus_^−1^], the pulse duration *tp* [µs], the frequency *f* [Hz], and the pulse number *n* [−]; n.r. stands for not recorded, HPH for high‐pressure homogenization, DW for dry weight, US for ultrasonication, CH for treatment chamber geometry, and PS for pulse shape.

^a^ PEF combined with organic solvent systems

Protein and carbohydrate recovery have been demonstrated in microalgae and cyanobacteria under continuous‐flow conditions. In *Chlorella vulgaris*, PEF enabled protein and carbohydrate recoveries of 5.2% and 35.8% relative to a high‐pressure homogenization‐defined extractable fraction (Carullo et al. 2018). In *A. platensis*, water‐soluble proteins, carbohydrates, and C‐phycocyanin reached 25.4%, 64.1%, and 37.4%, respectively, using the same normalization approach (Carullo et al. 2020). Several studies indicated that post‐treatment incubation can dominate the observed outcome. In *C. vulgaris*, protein release reached 22% on a dry biomass weight basis after 24 h incubation, consistent with diffusion‐limited transport following permeabilization (Scherer et al. 2019; Krust et al. 2022), while in *Auxenochlorella protothecoides*, incubation conditions influenced lipid release during extraction (Delso et al. 2022). Similar concepts extend to yeast. In *Saccharomyces cerevisiae*, protein extraction yields of about 88% and 80% were reported after incubation (Ganeva et al. 2020; Marín Sánchez et al. 2025). Beyond proteins, yeast applications include recovery of antioxidants and autolysis‐related products. Glutathione yields ranged from 70% to 78% of total cellular glutathione after incubation (Berzosa et al. 2024).

Reversible electropermeabilization has been explored to enable selective extraction while maintaining viability, including microalgae systems such as *H. pluvialis* and *C. vulgaris* (Gateau et al. 2021; Buchmann et al. 2019). However, viability is often assessed by bulk regrowth, which does not resolve the fraction of viable permeabilized cells at the single‐cell level. This limits the interpretation of whether recovered proteins originate from viable, permeabilized cells or from inactivated cells during treatment. Studies using more discriminating viability assessments suggest that protein release from truly viable cells can be low, which motivates approaches that increase mass transfer while limiting irreversible damage (Bodénès et al. 2019; Perez, Weber, et al. 2025).

Lipid‐related applications often require higher energy input, longer effective treatment, or integration with downstream steps. In *A. protothecoides*, PEF has been combined with solvent‐assisted extraction and post‐treatment incubation to increase lipid recovery (Delso et al. 2022; Silve, Kian, et al. 2018). In *C. reinhardtii*, PEF combined with mechanical disruption increased lipid recovery relative to untreated samples (Bensalem et al. 2018). Outcomes are strongly organism‐dependent. A cell wall‐deficient strain of *C. reinhardtii* enabled lipid droplet recovery under moderate energy input in continuous‐flow conditions (Baumgartner et al. 2025). In contrast, *Chlorella sorokiniana* showed no significant lipid increase under the maximum PEF conditions tested, indicating that robust envelope structures can require more intensive treatment or additional steps (Leonhardt et al. 2020). PEF has also been applied to pigments and metabolites. Astaxanthin extraction has been reported for *Xanthophyllomyces dendrorhous*, and lutein extraction has been reported for *C. vulgaris* (Aguilar Machado et al. 2020; Luengo, Martínez, Bordetas, et al. 2015).

Cross‐study comparison remains limited by heterogeneous designs, missing reporting parameters, and inconsistent normalization. Some studies normalize yields to recoveries obtained using reference disruption methods such as high‐pressure homogenization, bead milling, or ultrasonication, as shown for *C. vulgaris*, *A. platensis*, *S. cerevisiae*, and *H. pluvialis* (Carullo et al. 2018, 2020; Ganeva et al. 2020; Gateau et al. 2021). Other studies report yields on a dry biomass basis or as relative change versus untreated controls, as shown for *C. vulgaris*, *X. dendrorhous*, and *A. falcatus* (Scherer et al. 2019; Aguilar Machado et al. 2020; Antezana et al. 2013). These differences complicate benchmarking and can obscure treatment effects. Scalable and translatable applications, therefore, require a reporting and design framework that links field delivery, including pulse width, pulse shape, polarity, and pulse number, to defined outcomes across organisms and process goals.

This requirement is most evident for applications that aim to tune permeability within a reversible window rather than to maximize cell disruption. For these applications, small changes in pulse delivery can shift the outcome from selective transport to irreversible damage, thereby altering selectivity, product purity, and viability. Pulse grouping and waveform strategies have therefore been proposed to increase permeabilization efficiency at constant specific energy input, thereby improving permeability control without adding heating or unnecessary damage (Pakhomova et al. 2013; Perez, Weber, et al. 2025; L. Li et al. 2025). In microalgae, these approaches have been evaluated in *A. protothecoides* to increase permeabilization at fixed energy input (Perez, Weber, et al. 2025). Comparable permeability control is central to applications such as improving lipid bioaccessibility in *C. vulgaris* and enabling intracellular uptake for mineral and vitamin enrichment in *S. cerevisiae* (Canelli et al. 2022; Nowosad et al. 2021, 2023).

## 5 Pulse Parameter Selection

Selecting appropriate pulse parameters is crucial to maximizing permeabilization by PEF treatments while minimizing undesired effects, such as excessive heating or electrode degradation (Martínez et al. 2020). Pulse characteristics directly influence the mechanism and extent of membrane permeabilization, as described in Section 3, and their selection must align with the intended bioprocessing goal, whether it is reversible permeabilization for compound delivery or irreversible disruption for compound extraction.

Pulse duration is one of the most critical parameters, determining the biophysical target of the treatment, and can range from nanoseconds to milliseconds. Nanosecond pulses (nsPEF, < 100 ns) are typically shorter than the membrane charging time, which limits conventional electropermeabilization but allows *E* to interact with intracellular structures (Haberkorn et al. 2021). Under appropriate experimental conditions, however, sufficiently strong *E* can still induce membrane permeabilization, even within the nanosecond range, but with smaller permeable sites that lead to reduced mass transfer (Gianulis et al. 2018). nsPEF pulses have been associated with various physiological responses, including stress adaptation, signal modulation, and cell differentiation, as reviewed in detail elsewhere (Haberkorn et al. 2021).

Millisecond pulses (msPEF, <5 ms) can induce membrane permeabilization due to their extended duration, which exceeds the time required for membrane charging. However, their application often results in substantial Joule heating. It has also been reported that ms pulses have a lower permeabilization efficiency and reduced inactivation capacity, compared to microsecond pulses (Luengo, Martínez, Coustets, et al. 2015). These limitations restrict their use in most bioprocessing contexts. msPEF may still be applied in systems with very low electrical conductivity, where heating is less pronounced, or in cases where the PEF generator has technical limitations, such as a maximum voltage limit, that prevent the application of shorter, more intense pulses. Moreover, some studies have suggested that longer pulses facilitate the transfer of larger molecules, such as DNA, by providing stronger electrophoretic forces that support the attachment of charged molecules to the cell for transport (Rols and Teissié 1998).

In contrast, microsecond pulses (µsPEF, <500 µs) provide sufficient duration for complete membrane charging. This enables the formation of stable hydrophilic pores and large permeabilized areas that facilitate mass transfer (Navickaite et al. 2020). This pulse range is adaptable, allowing for both reversible and irreversible permeabilization, depending on the applied *E*, pulse number, and waveform. Due to its balance between efficiency, selectivity, and manageable thermal effects, µsPEF has become the predominant modality in PEF‐based bioprocessing, commonly applied in various biological systems (Figure 2; Luengo, Martínez, Coustets, et al. 2015; Martínez et al. 2020; Navickaite et al. 2020).

### 5.1 Interplay Between Pulse Duration and Electric Field Strength in µsPEF

Depending on the processing goal, different *E* are most effective. For irreversible electropermeabilization, higher *E* values, far above *E* needed to reach *ΔΨ_crit_*, are generally preferred (Knappert et al. 2020; Scherer et al. 2019), as they can generate larger or more pores (Pakhomov et al. 2007; Silkunas et al. 2024), facilitating the release of intracellular compounds. In certain microorganisms, these conditions can additionally weaken or disrupt the cell wall, improving mass transfer (Bensalem et al. 2020; Ganeva et al. 2014). However, prolonged exposure to high fields increases the risk of excessive heating, which can cause thermal damage and loss of selectivity in extraction (Marín‐Sánchez et al. 2025; Timira et al. 2022).

Reversible electropermeabilization, in contrast, works best with moderate *E* values applied for longer durations (Rols and Teissié 1998). These conditions promote the formation of transient yet stable pores, enabling molecular transport without irreversible structural damage (Najim and Aryana 2013; Perez, Weber, et al. 2025). Nevertheless, the transport of macromolecules involves both electrophoretic forces and biological interactions between membranes and molecules (Demidchik et al. 2018; Rols and Teissié 1998). Thus, choosing the appropriate combination of field strength and pulse duration is imperative and application‐specific, with several combinations possible (Figure 2). Generally, short, high‐intensity pulses are more suitable for efficient extraction, while longer, moderate‐intensity pulses are preferable for reversible treatments.

### 5.2 Pulse Shape

**FIGURE 3:**
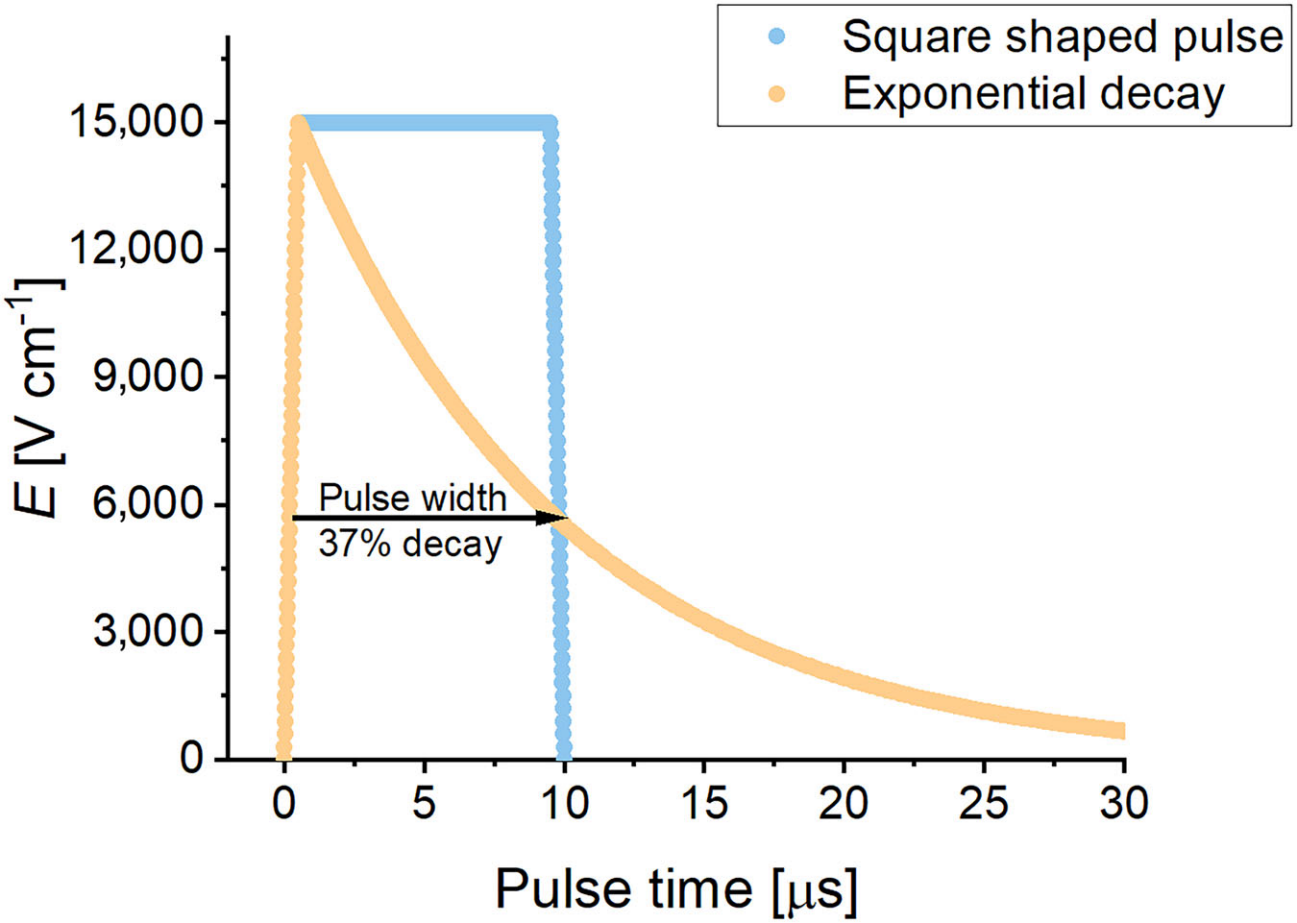
Schematic representation of square and exponential decay electric field pulses over a 10 µs duration. For the exponential pulse, the pulse length is defined as the time required for the field to decay to 37% of its initial peak value, corresponding to one time constant (τ). The rise time is assumed to be 0.5 µs for both pulse types. The exponential pulse discharges following the typical decay of an RC circuit, but it can be halted at a specific time depending on when the generator turns off the voltage. A constant electrode distance of 0.4 cm is assumed in the chamber for this graphical representation.

Two main types of electric pulses are commonly used in PEF applications: exponential decay pulses and square (rectangular) pulses (Figure 3). In square pulses, *E* is maintained at a constant level for a defined duration, meaning the observed effects result directly from the specific applied *E*. With exponential‐decay pulses, however, the voltage decreases continuously, and the practical outcome can depend on the levels of *E* experienced by the cells over time (Knappert et al. 2020), with only a short duration spent at the maximum field. Both square and exponential decay pulses can be applied in unipolar or bipolar configurations. Bipolar pulses reduce electrochemical reactions and minimize electrode fouling, especially at the anode (Straessner et al. 2016). However, depending on the pulsing conditions, bipolar pulses can also produce a reduced effect due to bipolar cancelation (Tang et al. 2022), and producing such pulses requires more complex circuits and generators.

### 5.3 Split Pulse and Fractionated Dose Effects: Electrosensitization and Desensitization

Electrosensitization and desensitization are timing‐dependent effects that arise from pulse grouping in PEF protocols. Desensitization typically occurs when many pulses are delivered simultaneously as a continuous train. Early permeabilization increases membrane conductance (Silve et al. 2014), thereby reducing membrane charging and lowering *ΔΨ_ind_* during later pulses. The incremental effect per pulse therefore decreases, and additional pulses mainly add energy and heat rather than further permeabilization or inactivation (Muratori et al. 2017). This trend is supported by modeling showing reduced pore formation across successive pulses (Son et al. 2016) and by microbial inactivation curves that exhibit tailing at high pulse numbers, indicating diminishing inactivation per pulse (Delso et al. 2020).

In contrast, electrosensitization describes an increased susceptibility to PEF following an initial PEF exposure and is often associated with fractionated delivery, in which a pulse train is divided into segments separated by pauses. Split treatment was introduced in biomedical electropermeabilization (Pakhomova et al. 2011, 2013) and has been shown to improve mass transfer and inactivation. However, the mechanisms underlying this effect remain poorly understood (Muratori et al. 2017). In single‐cell systems, split treatment helped overcome tailing in *Salmonella* Typhimurium, where longer inter‐treatment intervals, up to hours, improved inactivation, and the optimal timing depended on organism and medium properties, including pH (Delso et al. 2020).

In bioprocessing, pulse train fractionation can therefore reduce desensitization within the train and enhance electropermeabilization. Strategies include splitting longer pulses into shorter ones at low repetition rates, which has been shown to enhance permeabilization and protein release in microalgae (Perez, Weber, et al. 2025). Moreover, increased permeabilization and extraction have been reported in microalgae treated with an oscillating superimposed rectangular pulse (L. Li et al. 2025). An oscillating pulse can be viewed as a form of intrapulse segmentation, as it contains repeated polarity changes that act as successive subpulses. In continuous flow, generator limits and short residence times can constrain split‐based protocols, so staged treatment zones offer a practical approach to fractionated exposure while maintaining stable flow.

Overall, desensitization is expected when pulses are applied without breaks. In contrast, electrosensitization can be promoted when pulses are split, or the train is divided into segments with sufficiently long pauses to allow recovery.

## 6 Physicochemical Interactions With the Medium

The composition and properties of the treatment medium significantly influence the outcome of PEF treatments. The following subsections highlight the primary medium‐related factors that affect treatment efficiency.

### 6.1 Electrical Conductivity and Temperature

The electrical conductivity σ(T) will affect the energy input to the system, the Joule heating of the treated suspension, and the cells due to changes in the medium's osmolarity. Changes in the medium's conductivity can also alter the final voltage the generator can deliver (Buchmann et al. 2019); therefore, it is crucial to understand how these effects are related. The conductivity is dependent on the temperature σ(T) [S m^−1^]. It is inversely related to the resistance *R* [Ω]. *R* depends on the electrode area *A* [m^2^], and electrode gap *L* [m] (Equation 6). According to Ohm's law, an increase in σ(T) will result in a decrease in *R*. For a constant voltage *U* [V], this decrease in resistance causes an increase in current *I* [A] (Equation 7).

(6) $$R=\frac{L}{\sigma \left(T\right)\cdot A}\;$$

(7) U=I·R

Current generates heat through Joule heating *Q* [J], which can be described as an energy input (Equation 8). The total heat produced depends on *I*, *R*, and the cumulative exposure time *t* [s], which is defined by the number of pulses and their duration. As the temperature rises, σ(T) creates a positive feedback loop that can further intensify internal heating. Joule heating develops rapidly and is difficult to dissipate during the treatment process.

(8) $$Q=I^{2}\cdot R\cdot t$$

Although experimental results vary, numerical simulations have indicated that maintaining a moderate medium conductivity does not compromise the electropermeabilization of the cells (Ivorra et al. 2010). In contrast, very low conductivity, such as in distilled water, can hinder permeabilization and often requires higher *E* (Gianulis et al. 2018; Ivorra et al. 2010). Ultimately, maintaining low to moderate conductivity (>0.01, <10 mS cm^−1^) is recommended to reduce energy demands, avoid excessive heating, and prevent osmotic stress that may affect cell morphology and membrane charging (Ruzgys et al. 2019).

### 6.2 Temperature Effects

Temperature is an intertwined variable in PEF because it acts both as an input before PEF is applied and as an outcome of Joule heating. This heating can lead to axial and local temperature gradients in the treatment zone and may cause hot spots when the electric field and flow are uneven (Lindgren et al. 2002). At the membrane level, temperature affects the threshold for permeabilization; notably, the critical voltage for electropermeabilization rises sharply as temperature drops, roughly doubling when cooling from 37°C to 5°C, aligning with temperature‐dependent viscoelastic membrane properties (Di´az and Rubinsky 2004). In terms of process outcomes, higher initial or treatment temperatures generally improve microbial inactivation by PEF, especially when thermal stress is combined with pulsing for irreversible effects (Knappert et al. 2020; Nabilah et al. 2022; Timmermans et al. 2014). Temperature also impacts recovery and resealing, though effects vary system by system. For erythrocytes, increasing temperature at pulsing from 4°C to 43°C reduced hemolysis, while the resealing fraction and rate increased with both pulse and incubation temperatures (Nanda and Mishra 1994). For extraction and product recovery, monitoring and controlling temperature are crucial for distinguishing electric‐field effects from thermal contributions and preventing thermal degradation of the target compound.

### 6.3 Osmolarity

Osmosis is the movement of solvent across a semipermeable membrane driven by a solute concentration gradient, from the less concentrated to the more concentrated side. During PEF, medium osmolarity can modulate membrane behavior. Resuspension in a medium with higher osmolarity, compared to the medium after PEF treatment has been shown to enhance membrane resealing and improve post‐treatment cell recovery in yeast, whereas lower osmolarity favors compound release (Gančytė et al. 2023). For reversible electropermeabilization and cell survival, maintaining an osmolarity close to that of the growth medium is recommended (H. Wang and Lu 2006). However, when the goal is extraction, low osmolarity may improve compound diffusion but also risks uncontrolled cell lysis (Poblete‐Castro et al. 2020), potentially compromising downstream separation processes.

### 6.4 Water Dissociation Equilibrium and pH

Changes in the balance between H^+^ and OH^−^ ions during PEF treatment can influence cellular responses. In non‐extremophile organisms, low pH conditions, associated with elevated H^+^ concentrations, may disrupt intracellular pH regulation and reduce cell viability (Saldaña et al. 2010). At the same time, acidic conditions can promote autolysis and improve compound extraction during post‐treatment incubation (Delso et al. 2022).

Although pH values near neutrality are often preferred to preserve cell integrity, local pH shifts can occur near the electrodes due to electrochemical reactions involving water. These changes in ion concentrations are more pronounced in microchannel systems because of limited diffusion, which restricts the equilibration of local pH (Y. Li et al. 2015). This effect may alter the behavior of pH‐sensitive compounds and should be considered when targeting molecules such as proteins (Axelrod et al. 2023).

## 7 Biological Factors influencing PEF Response

The efficiency of electropermeabilization treatments can be strongly influenced by biological barriers at the cellular level, which govern membrane permeabilization and mass transfer. Moreover, indirect biological effects triggered by PEF treatments, such as autolysis and cell wall degradation, are also present. While extensive work has been done on non‐extremophile microorganisms, revealing species‐dependent differences in PEF susceptibility, emerging interest has shifted toward extremophiles due to their robustness and potential as biofactories (Neifar et al. 2015). This section discusses the key cellular features that limit or enable mass transfer after PEF exposure, with emphasis on how the unique physiological traits of extremophiles challenge conventional treatment strategies.

### 7.1 Species‐Specific Biological Barriers Influencing PEF Response

The PEF processing outcome can be highly dependent on species‐specific cellular characteristics, particularly the structure of the cell envelope and the organism's physiological state (Aguilar‐Machado et al. 2020; Bensalem et al. 2018; Polak et al. 2014a, 2014b). These biological characteristics determine a cells’ susceptibility to electropermeabilization and mass transfer efficiency; therefore, they must be considered during process design. For example, membrane fluidity is a dynamic property. Organisms adjust their membrane fatty acid profiles to maintain fluidity under temperature stress through a process known as homeoviscous adaptation. This process involves increasing the unsaturation or branching of lipid chains when the temperature decreases (L. H. Wang et al. 2016; R. Y. Wang et al. 2019). These changes enhance membrane fluidity and higher unsaturation, making cells more susceptible to pore formation during PEF since initial pore formation is relatively faster and more lipid oxidation can occur (Ernst et al. 2016; L. H. Wang et al. 2016). However, PEF can also induce structural responses, such as decreased fluidity or the activation of membrane repair mechanisms. In *S*. Typhimurium, reduced membrane fluidity was observed after PEF exposure (Yun et al. 2016), and in *E. coli*, genes related to membrane biosynthesis were upregulated following treatment (Kuang et al. 2024).

**FIGURE 4:**
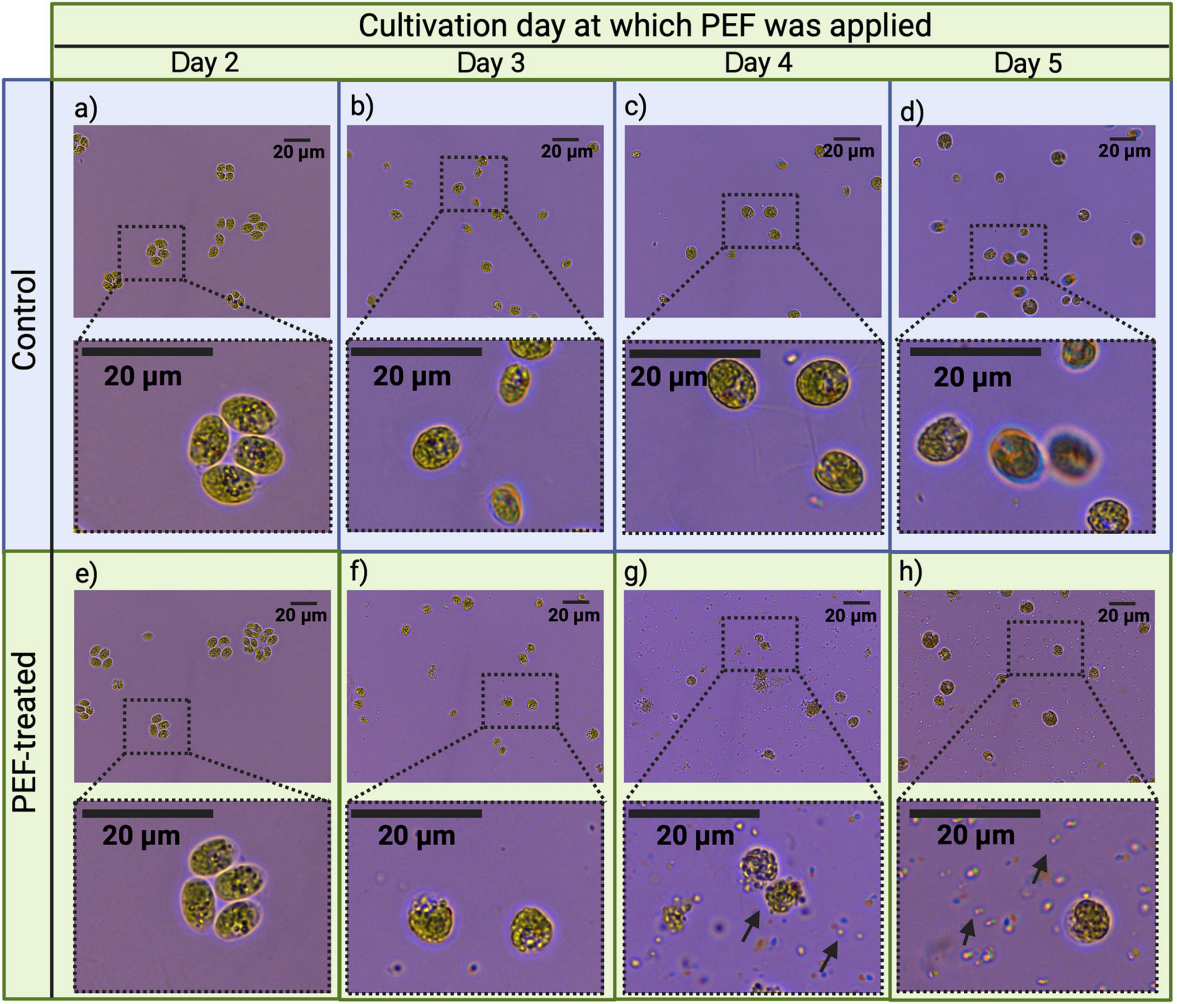
Effect of physiological state on PEF‐induced cell disintegration in *Chlamydomonas reinhardtii*. Bright field microscopy images of *C. reinhardtii* WT12 during batch cultivation, comparing untreated controls (a to d) and PEF treated samples (e to h) on days 2 to 5. Cultures were grown under conditions consistent with Baumgartner et al. 2025 and Pavlin et al. 2002 (inoculation OD750 0.2, constant light 168 ± 12 µmol m^−2^ s^−1^, 5% CO_2_, 150 rpm, 100 mL of autotrophic medium with 6.2 mM urea in a 500 mL Erlenmeyer flask). PEF was applied daily at *E* 14.2 ± 0.7 kV cm^−1^, pulse width 4.6 ± 0.0 µs, frequency 1 Hz, and 9 pulses, followed by imaging. Insets show magnified regions; scale bar 20 µm. Black arrows in panels g and h indicate representative disrupted cells and released intracellular material. The arrows are sized for visibility and do not represent a physical dimension. On Day 2, cells are mainly in an asexual reproduction phase and occur as clusters, and no clear disintegration is visible after PEF in bright field images, although clustering may still influence treatment outcomes. By Day 3, cells are mostly dispersed, and PEF still does not produce visible disintegration. After approximately Day 4, nitrogen limitation is expected, and gametogenesis may be induced in a subpopulation, a state associated with reduced wall integrity or wall loss. Under these conditions, PEF leads to visible cell disintegration on Days 4 and 5. These observations indicate that extraction and selectivity are expected to vary strongly with cultivation day and physiological state.

Although PEF primarily acts on the plasma membrane, it can also affect the cell wall and influence treatment outcomes. Cell wall structural characteristics vary significantly across species and growth conditions, thereby affecting mass transfer during and after permeabilization. For instance, *C. reinhardtii* changes its morphology and cell wall layers during growth, especially if nutrient availability changes, which can affect its response to PEF (Figure 4). In *C. vulgaris*, which forms denser walls during later cultivation stages, it has been shown to reduce the protein yield (Buchmann et al. 2019). These cell wall adaptations are typically viewed as diffusion barriers; however, Bensalem et al. (2018) reported improved extractability from stressed *C. reinhardtii* cells, suggesting that wall thickening alone does not determine extraction efficiency. Instead, the physiological state and solvent interactions likely play a more decisive role. Structural changes in the wall may also arise directly from PEF exposure. In *C. reinhardtii*, PEF treatment increased permeability to 3 kDa dextrans and enabled diffusion of up to 70 kDa at higher intensities (Bensalem et al. 2020). Similar enhancements in wall porosity were observed in yeast, as evidenced by increased lyticase activity and permeability to specific cell wall‐impermeable molecules (Ganeva et al. 2014; Stirke et al. 2019).

### 7.2 Indirect Biological Effects Triggered by PEF

PEF can induce delayed biological responses that contribute to extraction during post‐treatment incubation, beyond the immediate increase in membrane permeability. In microalgae, incubation after PEF has enhanced the release of intracellular compounds, including lipids in *A. protothecoides* (Delso et al. 2022) and proteins in *C. vulgaris* and *A. protothecoides* (Perez et al. 2024; Silve, Kian, et al. 2018). In *C. vulgaris*, lipid bioaccessibility also increased without compromising oxidative stability (Canelli et al. 2022). Similar effects have been reported in yeast. In *S. cerevisiae*, post‐treatment incubation promoted the recovery of glutathione and proteins, consistent with autolysis‐associated release after PEF (Berzosa et al. 2024, 2025). These outcomes are commonly linked to endogenous enzymatic activity and autolytic processes that gradually weaken the cell wall and envelope, facilitating mass transfer without complete rupture (Canelli et al. 2022; Perez et al. 2024; Berzosa et al. 2025). Such controlled release can reduce fine debris formation and support downstream separation performance (Perez, Li, et al. 2025).

The effectiveness of post‐treatment incubation depends on the incubation environment and the cells’ physiological state. Reported drivers include medium pH, which can modulate wall weakening and enzymatic activity (Delso et al. 2022; Berzosa et al. 2024), as well as metabolic status and growth phase, which influence the capacity for enzyme‐mediated activity during incubation (Krust et al. 2022). These factors require systematic evaluation when post‐treatment incubation is used as a process lever to improve mass transfer.

In bacteria, the role of post‐treatment holding is typically different from that in microalgae and yeast. It is mainly discussed in terms of injury and recovery, not as a strategy to enhance extraction. García et al. (2005) reported sublethal injury that depended on treatment intensity and medium pH in gram‐positive species such as *Bacillus subtilis* and *Listeria monocytogenes*, and in gram‐negative species including *E. coli*, *Pseudomonas aeruginosa*, *Salmonella* Senftenberg 775 W, *S*. Typhimurium, and *Yersinia enterocolitica*. This indicates that post‐treatment time can reflect envelope repair and resealing, which can reduce permeability and complicate extraction‐related interpretation if holding conditions are not controlled and reported. In *S*. Typhimurium, Delso et al. (2020) analyzed tailing in PEF inactivation curves, consistent with a time‐dependent change in susceptibility during post‐treatment periods. Together, these observations suggest that the controllable window between transient permeabilization and loss of culturability can be narrow in bacteria, in part because smaller cells typically require higher electric fields than larger cells to reach comparable induced transmembrane potentials (Choi et al. 2022).

### 7.3 Specific Challenges in Extremophiles

Extremophile organisms are rich in industrially relevant bioactive compounds and enzymes, often possessing unique properties not found in non‐extremophile systems. In food bioprocessing applications, acidophiles or thermophiles are relevant due to their reduced contamination levels, heat resistance, and acid tolerance (Gomes et al. 2016; Wan et al. 2021). For example, the C‐phycocyanin produced by *Galdieria sulphuraria* has been recently approved as a color food additive (FDA 2025). The extract has good acid stability and, therefore, offers the possibility of use in beverages as a natural colorant (Wan et al. 2021). The phylogenetic diversity of extremophiles across eukaryotes, prokaryotes, and archaea indicates a wide range of novel compounds and ‘extremozymes’. They are often highly resistant to extreme environmental factors (cold‐stable, thermoactive, oxidant‐stable) encountered in food processes (Mutlu‐Ingok et al. 2022) and are regarded as underutilized in food and bioprocessing applications (Neifar et al. 2015).

The application of PEF to extremophilic organisms faces challenges. Sommer et al. (2021) were unsuccessful in phycocyanin PEF extraction from acidophile/thermophile *Cyanidium caldarium*. Simulations of hyperthermophilic membranes of the archaeon *Aeropyrum pernix* demonstrated an increase in the electric field strength to reach the critical membrane potential (Polak et al. 2014a, 2014b). Extremophiles, which thrive in high temperatures or acidic environments, typically have rigid, tightly packed membranes. This is due to specialized lipids that contain ether and tetraether bonds, which increase stability and reduce deformability (Polak et al. 2014b). Even when the *ΔΨ_ind_* is high, the mechanical resistance of the membrane may prevent efficient pore formation. Computational studies have shown that introducing mesophilic non‐extremophile lipids into hyperthermophilic archaeal membranes lowers the *ΔΨ_crit_* required for pore formation (Polak et al. 2014b), highlighting the importance of membrane composition in electroporation outcomes.

The second challenge concerns the electrochemical properties of extremophile membranes (Matin 2010). Organisms living in highly osmotic or acidic environments maintain steep ionic gradients across their membranes. While the intracellular pH typically ranges from 5.5 to 6.8, external pH values can be as low as 0.06 (Rampelotto 2013). Due to the permeability of membranes to protons and the high energetic cost of active cation extrusion, acidophiles frequently accumulate intracellular cations. This results in *ΔΨ_rest_* that are either low or even positive (Matin 2010). Similarly, halophiles manage osmotic stress by utilizing intracellular potassium and compatible solutes, which can occasionally lead to collapsed or inverted membrane potentials (Oren 2013). These phenomena are described by the Nernst equation solved for the resting transmembrane potential (Equation 9):

(9) $$\Delta \Psi_{\mathrm{rest}}=\Delta p+\frac{2.3\cdot \mathrm{Rg}\cdot T}{F}\Delta \mathrm{pH}$$

where the proton motive force Δp [V] across acidophilic membranes determines the resting membrane potential *ΔΨ_rest_* [V], the equation considers the pH difference across the membrane ΔpH, the universal gas constant *Rg* [J mol^−1^ K^−1^], absolute temperature *T* [K], and the Faradays constant *F* [C mol^−1^].

Deviations from the typical resting transmembrane potential (*ΔΨ_rest_* ≈ −200 mV), which are common in non‐extremophile organisms, can significantly influence how the induced transmembrane potential *ΔΨ_ind_* combines with the intrinsic *ΔΨ_rest_* to form the *ΔΨ_total_*, as shown in Equation 2. Specifically, in some regions of the cell membrane, such as the side facing the anode, the *ΔΨ_total_* may be decreased. This decrease can prevent the membrane from reaching the critical threshold needed for electroporation, particularly at low applied voltages. However, this electrostatic interaction alone does not fully explain the observed differences in membrane rupture (Polak et al. 2014b; Sommer et al. 2021) since even the highest reported *ΔΨ_rest_* values (up to +120 mV; Matin 2010) remain considerably lower than the typical *ΔΨ_ind_* achieved during standard PEF treatment *ΔΨ_ind_* > 1000 mV (1 V).

Recent experimental evidence indicates that *ΔΨ_rest_* not only influences the *ΔΨ_total_* but also modulates the mechanical properties of the membrane. Wadud et al. (2023) showed that a more negative *ΔΨ_rest_* increases lateral membrane tension, which, combined with the tension from the applied *E*, lowers the energy barrier for pore formation and enhances electropermeabilization efficiency. In contrast, extremophiles often exhibit less negative or even positive *ΔΨ_rest_* resulting in lower intrinsic tension and a higher threshold for membrane disruption, even when the *ΔΨ_crit_* should have been theoretically reached. Therefore, the effective application of PEF in extremophilic organisms requires consideration of both their electrochemical properties and the mechanical rigidity of their membranes, not only due to lipid composition but also *ΔΨ_rest_*. Future research should extend beyond voltage thresholds to investigate how membrane mechanics and tension impact electropermeabilization in these resilient systems.

This section emphasizes tailoring PEF parameters to the biological traits of the target species rather than assuming a universal cellular response. While predictions can be well‐informed, small variations in cultivation and treatment conditions may impact cell structure, including cell size, wall composition, membrane fluidity, and tension. Consequently, screening is advisable when testing new species or when physiological states vary.

## 8 Fast Screening of Electropermeabilization

A screening of treatment conditions is crucial to identify the optimal parameters for a specific application. There are three primary techniques, all based on tracer molecules: fluorescent dyes that indicate permeabilization, changes in the conductivity of the cell suspension, and alterations in cell electrical properties.

### 8.1 Fluorescent Staining

Several dyes are available for assessing membrane permeabilization, with propidium iodide (PI) and Sytox Green (SG) being among the most widely used. These cell‐impermeable dyes do not enter the cells unless the membrane is compromised, making them effective indicators of permeabilization. Once inside, they bind nucleic acids and increase their fluorescence several‐fold, helping distinguish compromised cells from non‐permeabilized ones (Lebaron et al. 1998; Thakur et al. 2015; Zand et al. 2022). Both PI and SG exhibit high fluorescence intensity; however, PI is more cost‐effective, whereas SG offers better penetration and mass transfer performance across various species. Additionally, their emission wavelengths differ and should be checked for potential interference with autofluorescence (Thakur et al. 2015; Zand et al. 2022). Optimization of the staining protocol is essential, including incubation time and, in some cases, temperature. Excessively high dye concentrations may lead to false positives by allowing penetration into intact cells, while too low concentrations may result in undetected permeabilization (Haberkorn et al. 2021). Using a sensitivity index can help to determine the correct dye concentration (Siliakus et al. 2017). Positive controls are commonly produced by heat‐treating, freeze–thawing, or chemically inactivating cells (Knappert et al. 2020; Perez, Li, et al. 2025).

Stained cells can be visualized using fluorescence microscopy; however, flow cytometry offers superior resolution and statistical power for high‐throughput analysis (Zand et al. 2022). As observed in forward‐ and side‐scatter plots, microalgae and yeast can be reliably gated based on their characteristic size and granularity. In microalgae, chlorophyll autofluorescence provides an additional gating parameter, allowing for a clear distinction between microalgae cells and background or cell debris (Haberkorn et al. 2021). Alternatively, fluorescence plate readers can be used for bulk measurements, though they lack single‐cell resolution and provide only an overall fluorescence intensity.

Although these methods are versatile, their effectiveness may be limited by cell‐specific properties or by medium conditions such as pH and buffer composition (Lebaron et al. 1998; Zand et al. 2022). It is important to note that the proportion of stained cells does not always correlate with extraction efficiency (Pavlin et al. 2005; Perez, Li, et al. 2025; Perez, Weber, et al. 2025). Alternatively, other mass transfer‐based methods, such as conductivity or impedance, can be used (Pavlin et al. 2005).

### 8.2 Conductivity Measurement

Measuring the suspension's conductivity with standard conductivity probes can be an efficient way to indicate overall ion leakage, which correlates with membrane disruption (Carullo et al. 2020; Pataro et al. 2011). It can be measured by an external conductivity meter or with the PEF setup attached to a voltage and current probe. As some ions may enter the cell, a controlled buffer suspension should be used, and results should be interpreted cautiously (Pavlin et al. 2005). A disruption index can be derived from conductivity changes to compare the efficiency of different treatment conditions (Carullo et al. 2018).

### 8.3 Impedance Analysis

Impedance‐based techniques evaluate the electrical properties of cell suspensions to detect changes in membrane integrity. At low frequencies, intact membranes function as capacitors, preventing current flow, whereas damaged or porous membranes allow current passage. At higher frequencies, the membrane's resistance decreases, permitting current to reach the cytoplasm even in healthy cells (Bürgel et al. 2015; Yang 2008). This frequency dependence makes impedance a valuable tool in PEF analysis for distinguishing between intact, permeabilized, and damaged cells. Measurements are often performed in low‐conductivity media, such as deionized water, to improve sensitivity (Yang 2008). However, it is important to consider that cells sensitive to low osmotic environments may burst under these conditions. In microfluidic channels, single‐cell impedance spectroscopy provides high‐resolution information on membrane status (Surowiec and Scholz 2023). Accurate detection requires careful frequency selection and consideration of the chemical reactions that are exacerbated in such small channels, such as water dissociation (Bürgel et al. 2015; Yang 2008).

Impedance also has other innovative applications, such as impedance flow cytometry, which can provide a label‐free method for quantifying viability and PEF‐induced permeability at the single‐cell level without the need for staining (Opitz et al. 2019). Moreover, in‐line sensors are being developed and have been integrated into fermentation setups to monitor cell viability (Liu et al. 2023), which could be used to track live‐cell permeabilization and enable closed‐loop optimization. However, these signals are indirect and susceptible to temperature drift, bubbles, electrode polarization, and fouling, necessitating robust probe design, temperature compensation, and periodic recalibration for routine industrial use.

Tracer‐based screening, such as with fluorescent dyes or ions, provides a good initial step for optimizing PEF conditions. However, application‐specific validation is necessary because the diffusion and transport kinetics of larger target compounds often differ from those of the tracers, especially at specific treatment and extraction media.

## 9 Engineering challenges

### 9.1 Cell Concentration

High cell concentrations are desirable in single‐cell bioprocessing because they increase throughput and reduce the volume that must be treated. This can lower energy demand for upstream concentration steps and reduce the total energy input required by the PEF system for a given production rate (Timira et al. 2022). However, dense suspensions exhibit higher viscosity and stronger non‐Newtonian behavior, which can alter residence time distributions and flow uniformity within treatment chambers. Monitoring and process control are therefore needed to maintain stable operation in continuous mode (Buchmann et al. 2018; Buckow et al. 2010; Perez, Azzari, et al. 2025).

Cell concentration can also affect the outcomes of electropermeabilization. In extraction‐oriented studies, concentrations above the commonly studied range of 5 g L^−1^ or below have often been associated with reduced yields, for example, in *C. vulgaris and A. protothecoides* (Scherer et al. 2019; Perez, Azzari, et al. 2025). This decrease can reflect hindered diffusion and solvent limitation, but it can also arise from electrical interactions between neighboring cells. Modeling showed that the induced transmembrane potential depends on cell density, arrangement, and position, which implies local field distortion and partial shielding at high volume fractions (Pavlin et al. 2002). In dense suspension experiments, permeabilization readouts based on dye uptake also decreased with increasing cell density, and higher applied fields were required to reach comparable permeabilization (Pucihar et al. 2007). A similar density dependence has been observed in bacteria. In *E. coli*, PI permeability decreased as optical density increased, for example, from 56.4% at OD 0.05 to 29.5% at OD 0.5 (Bar‐Hanun et al. 2025).

As biomass concentration increases, electrical conductivity can rise due to ions carried on the cell wall or intracellular ions released during PEF treatment, leading to Joule heating. In *E. coli* inactivation, higher bacterial densities boost conductivity and improve inactivation (Y. Wang et al. 2025). Conversely, some studies suggest that lower conductivity may enhance electropermeabilization (Silve et al. 2016), which could explain the varying results at different cell concentrations. These changes hinder reproducibility and interpretation, as reduced extraction may result from limited pore formation and weaker diffusion. Experimental validation for dense suspensions is crucial to confirm treatment effectiveness, and more research on mass transfer, medium interactions, and mixing effects is needed, as these influence whether low‐biomass trends are applicable at larger scales.

Components of the food matrix can weaken the effects of PEF and obstruct transport. For example, fat globules and proteins have been shown to decrease the inactivation of *Lactobacillus rhamnosus* in milk‐based systems, illustrating a protective matrix effect (Jäger et al. 2009). Soluble proteins and lipids can also hinder diffusion, complicate separation, and cause electrode fouling, reducing the effective treatment area (Axelrod et al. 2023). While it is not possible to alter the food matrix during inactivation studies, for single‐cell bioprocessing involving bioenrichment or extraction, for mass transfer applications, it is often advisable to harvest and resuspend cells in a well‐defined, low‐solute medium.

### 9.2 Treatment Chamber Design and Electric Field Distribution

**FIGURE 5:**
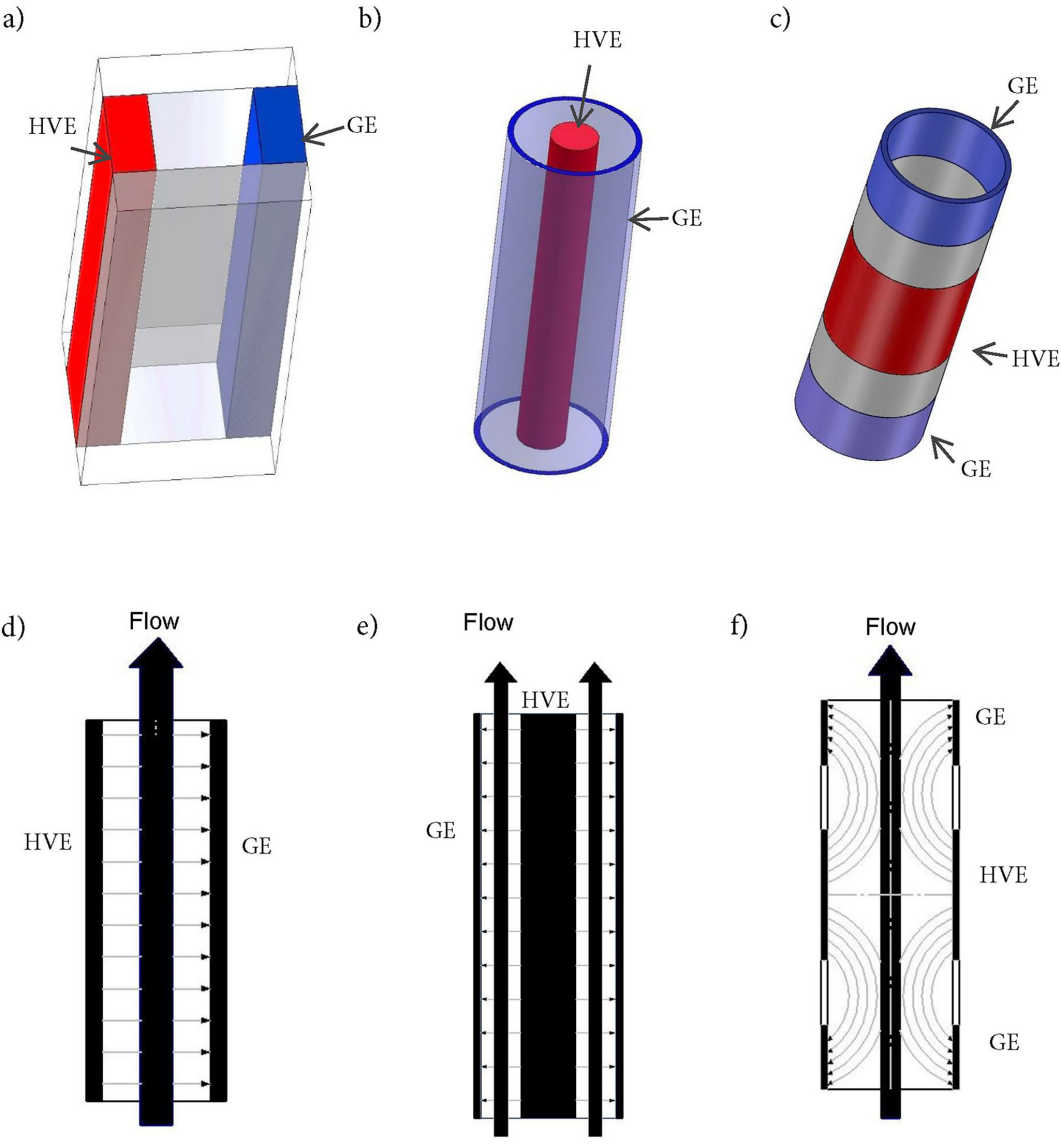
Schematic representation of common pulsed electric field (PEF) flow‐through chamber designs. (a, d) Parallel plate design with a uniform field across the channel. (b, e) Coaxial configuration with a central HVE and outer GE, creating a radial field. (c, f) Co‐linear setup with alternating HVE and GE rings generating a segmented axial field. Electrode arrangement influences field distribution, flow uniformity, and treatment efficiency. GE: ground electrode and HVE: high‐voltage electrode.

There are three main types of treatment chambers used in PEF processing: parallel plate (Figure 5a), coaxial (Figure 5b), and co‐linear chambers (Figure 5c). Each standard configuration has specific advantages and limitations as previously reviewed by Zand et al. (2021).

The parallel plate chamber generates a uniform *E* across the treatment zone, ensuring consistent exposure of cells to electric pulses (Buchmann et al. 2018; Figure 5d). This configuration typically features a square or rectangular cross‐section with relatively small dimensions promoting laminar flow, impacting residence time distributions. Sharp corners furthermore compromise hygienic design standards. However, proper engineering of the transition zones can reduce such effects and prevent fluid recirculation (Axelrod et al. 2023).

The coaxial chamber produces a close to homogeneous *E* (Figure 5e) and could therefore provide almost uniform treatment. Nevertheless, it is rarely used in single‐cell bioprocessing because of its manufacturing complexity, the greater risk of heating from large electrode surfaces in contact with the medium (Zand et al. 2021), and the potential complexity of cleaning due to the small gap between the two electrodes.

In contrast, the co‐linear chamber, although it does not maintain a constant *E* across the treatment zone (Figure 5f), is one of the most used configurations in the industry for liquid treatment due to the possibility of hygienic design, smaller electrode area, and consequently lower overall heating due to limited current flow. The field strength varies radially. Consequently, cells at different radial positions receive different field intensities (Knappert et al. 2020; Zand et al. 2021), which may lead to under‐ or overtreatment (exposure beyond the intended permeability window, where additional pulses add little mass transfer benefit) and local heating (hot spots). Recent designs aim to minimize these effects through computationally optimized geometries that improve field homogeneity and flow distribution (Yan et al. 2025). Other innovations, such as inserting grids or inducing controlled vortices, have also been proposed (Schottroff et al. 2020). However, these modifications can create difficult‐to‐clean areas, a critical consideration for food and sterile applications.

Across parallel plate, coaxial, and co‐linear chambers, further improvement should focus on reducing exposure variability by increasing field and flow homogeneity, tightening residence time distributions, and limiting temperature rise through pulse pattern and cooling strategies, while maintaining hygienic design and clean‐in‐place compatibility. Inline monitoring with upstream and downstream sensing can also support feedback control to compensate for changes in conductivity, temperature, and biomass concentration. For co‐linear chambers in particular, the main priority is mitigating the radial field gradient, for example, through staged or parallelized treatment sections and flow conditioning that improves radial mixing without introducing hard‐to‐clean features, while keeping electrode area low to limit local heating and electrode‐related effects.

### 9.3 Process Homogeneity and Residence Time Distribution

In continuous PEF systems, as in most continuous‐flow reactors, residence time is distributed rather than fixed because individual cells follow different flow paths and move through the treatment chamber at different speeds. This variability can lead to uneven exposure, with some cells receiving insufficient treatment and others receiving excessive treatment, which reduces process efficiency and reproducibility. The issue is particularly acute in dense or viscous suspensions, where flow behavior is heavily affected by biomass concentration and cell shape.

In small‐gap chambers operating under laminar flow, shear gradients across the channel lead to position‐dependent treatment intensities (Stickel and Powell 2005). At high biomass concentrations, elongated or irregularly shaped cells or mixed cell populations may align with the flow, altering suspension rheology and causing non‐Newtonian behavior, as observed in concentrated *A. platensis* cultures (Buchmann et al. 2018). Such rheological changes further broaden the residence time distribution and, therefore, the cells’ exposure to *E*.

Experimental methods such as colorimetric tracers, salt‐based particle tracking, or laser particle tracking can be used to determine residence time (Georget et al. 2013). Predictions of residence time distributions can also be generated using computational fluid dynamics (CFD). Rheological properties measured experimentally can be integrated into CFD simulations to uncover potential non‐uniformities at high resolution. This allows for adjustments to flow velocity, chamber design, or operating conditions before physical testing, thereby reducing costs (Axelrod et al. 2023; Perez, Azzari, et al. 2025). To verify these predictions and achieve an accurate depiction of flow behavior, ultrasonic velocity profiling (UVP) can serve as a supplementary validation method.

Using pulsed ultrasonic echography and Doppler shift detection, UVP measures spatiotemporal velocity fields along a measurement line with high spatial resolution (Takeda 1995). Its ability to operate in optically opaque suspensions, including dense single‐cell cultures, makes it suitable for verifying numerically simulated flow patterns and analyzing residence time distributions (Buchmann et al. 2018). UVP is less effective in small‐gap chambers but works best in tubes with an inner diameter larger than approximately 1 cm, where sufficient measurement depth allows for accurate velocity profile reconstruction. Comparing CFD‐derived velocity fields with UVP results enables assessing the model's accuracy and refining simulation parameters, thereby enhancing process reliability.

Integrating CFD modeling, rheological testing, and UVP validation provides a comprehensive approach to designing a chamber with a narrow residence‐time distribution. This is particularly vital when handling dense, highly viscous suspensions or mixed cell populations, where uniform treatment is crucial for reliable and scalable PEF performance.

## 10 Accounting for Energy and Reporting

Clear, detailed reporting of PEF parameters is needed to ensure reproducibility. Although the specific energy input *W_s_* [kJ kg_sus_^−1^] is the most frequently reported, this parameter alone cannot guarantee comparable treatment outcomes. *W_s_* is only meaningful for comparison when experiments are performed under similar conductivity conditions, with comparable pulse shapes that ensure the maximum voltage is applied for the intended treatment time, and at similar cell concentrations. Treatment conditions should be explicitly reported, including pulse shape, pulse width *t_p_* [s], number of pulses *n* [−], and electric field strength *E* [kV cm^−1^]. This level of detail allows for the treatment to be repeated accurately. For example, extensive details on reporting have been reviewed by Raso et al. (2016). Moreover, the physicochemical properties of the treatment medium, such as chemical composition, osmolarity, and electric conductivity, will also affect the final process outcome as explained earlier in this review. On the other hand, the way in which the process is delivered, that is, the type of pulse, treatment chamber design, batch, or continuous process, should also be described. The minimum reporting parameters are summarized in Table 2.

**TABLE 2:** Recommended reporting parameters.

Category	Parameter	Unit or format	Notes
**Minimum reporting parameters**			
Microbial system	Species and genus	Text	Report full name
	Microbial group	Microalgae, yeast, bacteria, etc.	For bacteria, report gram‐positive or gram‐negative when known
	Cell concentration at treatment	g L^−1^, cells mL^−1^	
Electrical treatment	Pulse shape	Square, exponential decay, other	Report polarity pattern if bipolar
	Electric field strength, *E*	kV cm^−1^	For non‐uniform fields, define the reported value, such as peak or volume averaged, and state how it was obtained.
	Pulse width, *t_p_*	µs, ns, ms	Use the time scale applied
	Number of pulses, *n*	Dimensionless	Report total delivery of pulse and if there was a special configuration of the pulse train
	Pulse repetition frequency, *f*	s^−1^, Hz	
Medium properties	Conductivity, *σ*	S m^−1^	Report before and after treatment if available
	Temperature	°C	Report the temperature before and after PEF treatment and specify the measurement position relative to the electrodes in the treatment chamber.
	pH	Dimensionless	Report before and after treatment if available
Process delivery	Treatment chamber design: type; gap *L;* treated zone volume *V_PEF_*	Parallel plate, colinear, coaxial, other; m; m^−3^	
	Process mode	Batch or continuous	Name cuvette or chamber model if relevant
	Volumetric flow rate, *F*	m^3^ s^−1^	Required for continuous mode. If batch, report treated volume instead.
Electrical monitoring	Applied voltage measurement *U* or *U(t)*	V	Measured waveform preferred. State if calculated or nominal
	Applied current measurement *I* or *I(t)*	A	Measured waveform preferred. State if calculated from conductivity
	Specific energy input, *W_s_*	kJ kg_sus_^−1^	State calculation basis and equation or reference
Extraction reporting	Analytical method	Text	
	Target compound yield	g L^−1^, kg kg^−1^	State the normalization basis
**Additional relevant reporting parameters**			
Microbial system	Strain	Text	
	Growth phase	Stationary, exponential, and so forth	
	Average size	µm	Report the mean diameter or equivalent diameter. For anisotropic cells, also report at least one additional dimension, such as length and width. Provide the standard deviation when available.
	Shape	Spherical, ellipsoidal, rod‐shaped or elongated	Note chain forming or aggregated cells when present
Electric treatment	Pulse rise time	ns to µs	
	Interpulse interval	ns to µs	Time between consecutive pulses, usually determined by the treatment frequency
	Interval between pulse trains	s to h	Break between train of pulses used in sensitization approaches
	Total treatment time (n⋅ *t_p_*)	s	This definition excludes the time between pulses and the interval between pulse trains. Rise time is typically negligible.
Medium properties	Osmolarity; ionic strength	mOsm kg^−1^; mol L^−1^	Measured or estimated
	Medium composition	g L^−1^	Salts, buffers, additives, and solvents.
Process delivery	Chamber factor; field mapping method	Text description; field line plot; or electric field map.	For non‐uniform electric fields
	Flow regime	Reynolds number	
	Residence time	s or distribution	Disclose the method for measuring or calculating
Post‐treatment handling	Incubation time and conditions	min,°C	Important for delayed permeabilization and extraction kinetics.
	Extraction details	Mixing and agitation, change of extraction medium, addition of solvents or adjustment of pH, and so forth	

Voltage and current values must be carefully monitored. In systems without automatic voltage adaptation, external measurement of the applied voltage is important because the output voltage deviates from the setpoint depending on the suspension conductivity and must be manually adjusted (Buchmann et al. 2019). Older PEF generators are particularly sensitive to conductivity changes, often showing voltage drops in high‐conductivity media due to increased current draw. Moreover, simultaneous measurement of voltage and current enables the calculation of specific energy input (*W_s_*) by integration (Equation 10; Raso et al. 2016). This formula applies to PEF delivery in batch mode, where a fixed treated volume *V_PEF_* [m^3^] and total number of pulses *n* [−] are known.

(10) $$W_{s}=n\cdot \frac{\int_{t_{0}}^{t_{p}}U\left(t\right)\cdot I\left(t\right)\;dt}{\rho \cdot V_{PEF}}$$

where the density of the suspension is *ρ* [kg m^−3^], and the pulse duration *t_p_* [s].

When integration is not feasible, for example, when only a single voltage and current value is available, and square pulses are applied, Equation (10) can be simplified to Equation (11). For other pulse shapes, such as exponential decay, integration is required because the voltage changes over time.

(11) $$W_{s}\approx n\cdot \frac{U\cdot I\cdot t_{p\;}}{\rho \cdot V_{PEF}}$$

If the current measurement is not available, equations requiring *I* cannot be applied. In this case, Equation (12) can be derived from Ohm's law (Equation 7), the definition of resistance (Equation 6), and the definition of *E* (Equation 1). The detailed derivation is available in the appendix of Perez et al. (2024).

(12) $$W_{s}=\frac{n\cdot E^{2}\cdot \sigma \cdot t_{p}}{\rho }$$

Equation (12) can be applied using the average conductivity measured before and after treatment, as conductivity usually increases after permeabilization. Accurate voltage measurement or automatic correction remains essential. This equation is valid only for systems with homogeneous *E*, such as parallel plate chambers and cuvettes (Buchmann et al. 2018). For co‐linear chambers, *E* distribution is non‐uniform, making nominal values misleading. In such cases, simulations or numerical approximations are necessary to estimate the effective field distribution. For this calculation, refer to Knappert et al. (2020) and Schottroff et al. (2020).

In continuous mode, *W_s_* can be expressed in the same form as in Equation (10) when a representative treatment volume *V_PEF_* and the pulse number are defined for the treatment zone. This volume can be obtained either from the treatment chamber geometry or estimated using Equation (13), provided the residence time *τ* [s] and the constant‐volumetric flow rate *F* [m^3^ s^−1^] are known. Because residence time is a distribution, a representative value, such as the mean or median *τ*, can be used, obtained using UVP, particle‐tracing methods, or simulations.

(13) $$V_{PEF}=F\cdot \tau$$

The number of pulses can be estimated as well with *τ* and the pulse repetition frequency *f* [s^−1^] (Equation 14).

(14) n=f·τ

The specific energy input can also be calculated directly without *τ* under steady operation, using *f* and *F*, which are typically known process variables (Equation 15).

(15) $$W_{s}=f\cdot \frac{\int_{t_{0}}^{t_{p}}U\left(t\right)\cdot I\left(t\right)\;dt}{\rho \cdot F}$$

While τ is not required to compute *W_s_*, it is required to describe exposure at the single‐cell level and to rationalize under‐ and overtreatment arising from residence time distributions, even when *W_s_* is held constant, as discussed in Section 9.3.

For extraction, in addition to reporting volumetric *W_s_* [J kg_sus_^−1^], reporting cell concentration [kg L^−1^] and compound yield [kg L^−1^] is essential: cell concentrations vary between experiments, with higher concentrations reducing the treated volume and thus the energy required per unit biomass. The product yield again depends on the cell concentration. Normalizing energy per yield, therefore, offers the most robust basis for comparing energy efficiency of extraction processes. However, energy should always be reported alongside cell and yield concentrations to allow flexibility and transparency for all future use of the data. Specific energy calculations are presented in Perez et al. (2024).

In a continuous system, the total energy delivered during processing, *W_total_* [KJ], should also be reported separately from the specific energy input, *W_s,_* to avoid confusion. *W_total_* can be obtained by multiplying *W_s_* by the mass of the treated suspension. This mass is obtained by multiplying the constant volumetric flow rate *F* and density *ρ*, and the total processing time *t_proc_* [s] (Equation 16).

(16) $$W_{total}=W_{s}\;\cdot \left(\rho \cdot F\cdot t_{proc}\right)$$

## 11 A Practical Approach to Electropermeabilization Research in Single‐Cell Applications

**FIGURE 6:**
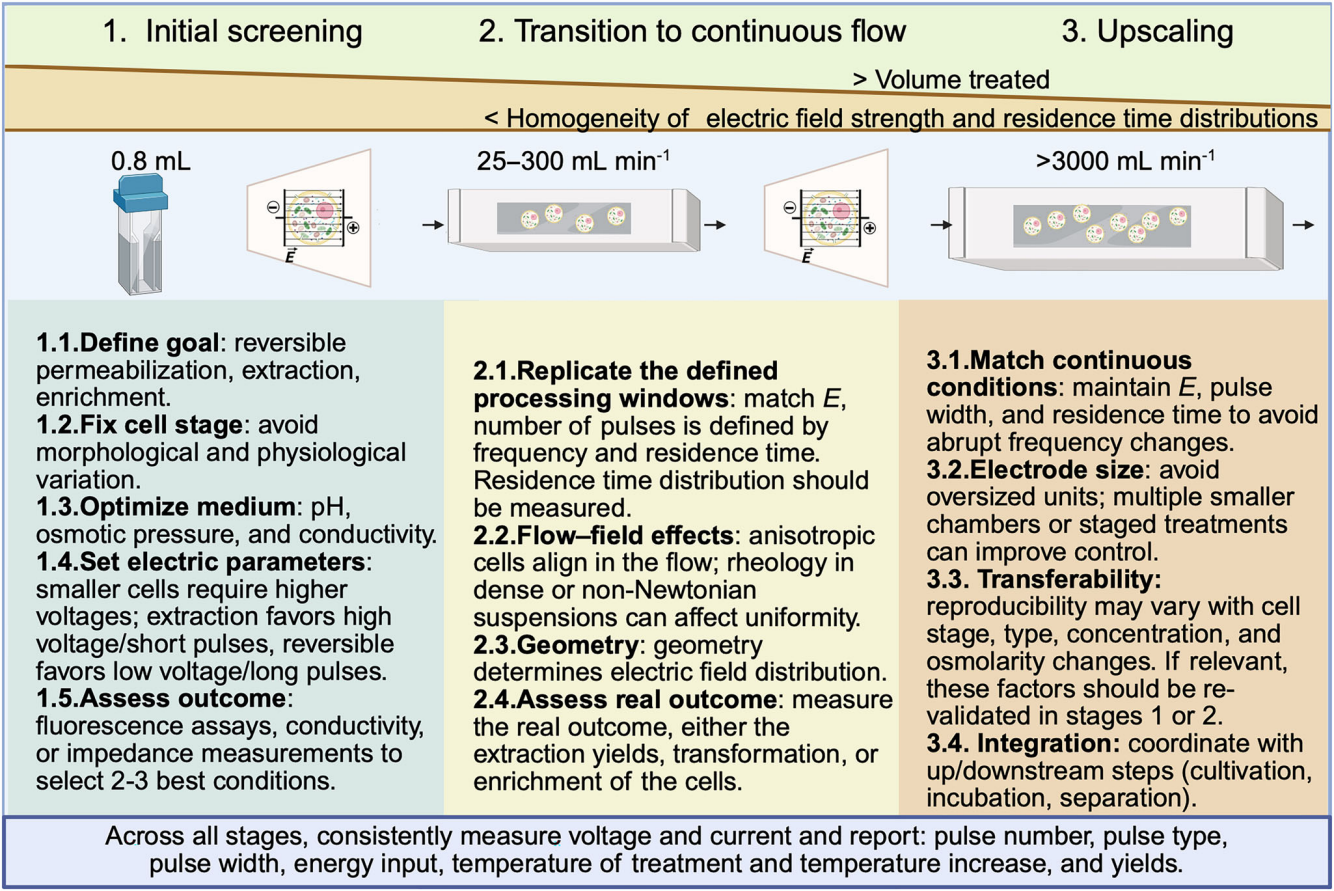
Workflow for electropermeabilization: from initial screening to continuous flow and upscaling. The process is divided into three stages: initial screening, transition to continuous flow, and upscaling. Each stage involves adjusting electric field parameters, residence time, and flow conditions while accounting for cell state, medium properties, and chamber geometry. Consistent measurement of voltage, pulse settings, temperature, and flow is required to ensure comparability between stages.

In this section, practical considerations and a proposed workflow for experimentation and upscaling of PEF treatments in single cells are presented. As highlighted in this review, no universal set of optimal conditions exists; parameters must be customized to a particular organism, medium, and available equipment. However, the main recommendations aim to facilitate a smoother transition between screening, continuous‐flow operation, and upscaling (Figure 6).

### 11.1 Initial Screening

1. Define goal. Clearly determine whether the target outcome is reversible permeabilization, compound extraction, or intracellular enrichment. The defined objective should guide the selection of medium composition and electrical parameters.
2. Fix cell stage. Ensure consistent cultivation to minimize morphological and physiological variability, unless the effect of growth stage is being investigated (Section 7).
3. Optimize medium (Section 6). Set and record the pH of the suspension. For reversible permeabilization, maintain a physiological pH in the electropermeabilization medium. For extraction applications, incubation at low pH (< 4) has shown favorable results (Delso et al. 2022; Perez et al. 2024). Osmotic pressure should also be considered: Lower osmolarity than the cultivation medium can facilitate extraction, whereas hypertonic conditions can promote resealing of permeabilized sites (Gančytė et al. 2023). Hypertonic conditions (osmolarity higher than in the original cultivation medium) can lead to complete leakage of cellular contents, depending on the cells’ susceptibility to osmotic stress. Osmolarity is typically correlated with electrical conductivity because dissolved ions contribute to it. In PEF treatments, low conductivity reduces energy input and heating. In terms of electropermeabilization efficiency, electrical conductivity seems to have little impact (Ivorra et al. 2010), although it is difficult to separate from temperature effects; however, impedance mismatch in the delivery system needs to be controlled (Buchmann et al. 2019). All parameters should be considered at the treatment‐specific temperature as discussed in Section 6.2. After electropermeabilization, the extraction of compounds from the cell will be governed by mass‐transfer mechanisms, particularly diffusion in the selected medium. For nonpolar compounds, different solvents are required.
4. Set the electric parameters (Section 5). The maximum voltage that can be applied depends on the generator capacity, the electrode spacing, and the medium's conductivity. High conductivity (>5 to 10 mS cm^−1^) at high *E* (>10 kV cm^−1^) can cause dielectric breakdown of the suspension and excessive current draw, potentially damaging both the generator and the cells. First, determine the maximum current output in your generator documentation (typically 100–300 A) and avoid exceeding this limit. Ensure that no sparking (dielectric breakdown) occurs, which can also happen when bubbles are present in the system. Smaller cells require higher voltages to reach the critical transmembrane potential, as explained in Section 3. High voltage can also permeabilize rigid cell walls and increase pore size (Bensalem et al. 2018; Ganeva et al. 2014; Silkunas et al. 2024). For extraction, high voltage with short pulses (**≈**2–20 µs) is recommended; for reversible permeabilization, low voltage with longer pulses (**>**100 µs) is used as explained in Section 5. Common combinations of electric field strength and pulse width are shown in Figure 2. Limit the specific energy input delivered during a single treatment to ≤100 kJ kg_sus_^−1^, which in water corresponds to a temperature increase of approximately 24°C. Exceeding this limit often results in thermal damage to the cells or target compounds. Cool down your suspension before treatment to prevent thermal damage to the cells or compounds.
5. Assess outcome. In the initial stages, easy approaches to evaluate permeabilization are using electroporation cuvettes in combination with fluorescence assays, conductivity measurements, or impedance analysis (see Section 8). Select the two to three best‐performing parameter sets within the established energy and temperature limits. If no continuous flow treatments are done, proceed to point 2.4.

### 11.2 Transition to continuous flow

1. Replicate the defined processing windows by matching the electric field strength, noting that it may vary with changes in the resistivity of the continuous‐flow chamber. Set the pulse number by adjusting the treatment frequency and the cell residence time in the chamber. Residence time is a distribution rather than a fixed value; the mean residence time can be used to define the required pulse frequency. Residence time distribution can be measured using UVP (Buchmann et al. 2018; Takeda 1995), tracers such as salt with conductivity sensors placed before and after the chamber (a cost‐effective and straightforward method; Georget et al. 2013), laser particle tracking, or colorimetric tracers. Residence time distributions can also be simulated based on the rheological properties of the cell suspension and validated using the aforementioned experimental methods (Axelrod et al. 2023; Perez, Azzari, et al. 2025), enabling a predictive, non‐experimental approach after validation. In some cases, plug flow can be assumed for small channels (Georget et al. 2013). For more details, see Section 9.3.
2. Flow–field effects. In laminar flow, anisotropic cells may align with the flow direction (Buchmann et al. 2018), thereby altering permeabilization efficiency by changing the cell surface area exposed to the electrodes. Rheological characterization provides insight into flow behavior, enabling improved homogeneity in the treated condition (Bernaerts et al. 2017; Perez, Azzari, et al. 2025). For suspensions with water‐like rheology, Newtonian behavior can be assumed.
3. Geometry. The geometry of the treatment chamber strongly influences electric field distribution and treatment uniformity. Co‐linear chambers generate irregular fields, resulting in non‐uniform cell permeabilization and variable extraction efficiencies across the population. In such cases, treatment effects can be approximated by integrating the total voltage delivered to the cells (Knappert et al. 2020). Alternatively, parallel‐plate chambers provide more uniform fields, enabling consistent yields and minimizing the risk of undertreated cells. When designing new chambers, particular attention should be paid to avoiding sharp edges and abrupt transitions, as these may induce fluid recirculation and overexpose specific cell regions (Axelrod et al. 2023). In practice, researchers should either evaluate the electric field distribution in existing chambers to understand treatment variability or design chambers with uniform geometries and confirm performance through simulations of the electric field distribution.
4. Assess real outcome. Measure actual yields for the target application (e.g., extraction, enrichment) to validate process performance. Screening results are less informative at this stage, as the molecule of interest may be too large to be accurately represented by tracer‐based permeabilization assays. Estimate the extraction success to ensure that an adequate sample volume is obtained to quantify the target compound.

### 11.3 Upscaling

1. Match continuous conditions. Maintain the validated electric field during upscaling, noting that it may change with increases in electrode area. If possible, retain the residence time by adjusting the length, width, and height of the upscaled chamber while increasing the flow rate. This approach prevents changes in pulse frequency that could lead to unexpected effects, such as insufficient time for cell resealing at very high frequencies (kHz domain) or generator limitations in delivering high energy within very short intervals.
2. Electrode size. Avoid using oversized electrodes, as they increase current draw and energy consumption, potentially leading to excessive heating and uncontrollable temperature rises. High energy inputs are likely under such conditions. For better process control, consider employing multiple smaller chambers or staged treatment sections.
3. Transferability. Reproducibility can be influenced by cell stage, cell type, concentration, and osmolarity. Revalidate key parameters during early trials whenever these conditions are expected to change at large‐scale volumes.
4. Integration. Ensure integration into the production process by aligning PEF treatment parameters with the requirements of both upstream cultivation and downstream processing. Consider spatial constraints, energy demand, and automation compatibility to facilitate efficient scale‐up.

### 11.4 Critical Control Points During Upscaling

During upscaling, reproducibility depends on confirming the delivered electric field with the correct pulse width, the actual number of pulses received per volume element under flow, and the temperature rise to avoid hot spots. These checks should be repeated when biomass concentration or medium conductivity changes, and when operating near generator current or duty cycle limits, or at frequencies above 1 kHz.

### 11.5 Across All Stages

Consistently measure and report at least the minimum reporting parameters specified in Table 2.

## 12 Conclusion

PEF technology provides a flexible approach to induce controlled membrane permeabilization in diverse single‐cell systems with minimal thermal load. Permeabilization results from both rapid electroporation and prolonged structural or biochemical changes, with efficiency shaped by cell wall structure, membrane composition, physiological state, and medium properties. No single set of processing conditions ensures the best performance; the optimal process depends on combining pulse parameters, medium formulation, generator capabilities, chamber geometry, and organism‐specific characteristics. Achieving high electropermeabilization efficiency and mass transfer rates, therefore, requires matching these factors to the specific constraints and goals of the application.

Future progress will benefit from quantitative studies on how cell concentration affects permeabilization and extraction in high‐density cell suspensions (> 90 g L^−1^) during continuous flow treatment. It will also include systematic characterization of extremophiles to determine their electromechanical thresholds and the integration of in‐line permeabilization measurement tools for immediate process adjustments. Combining these advancements with targeted strain selection and incubation protocols designed for specific extracted compounds can directly improve yield, selectivity, and energy efficiency. This improved process control and knowledge‐driven PEF design will also unlock the potential of emerging reversible permeabilization applications, such as nutrient enrichment and large‐scale targeted delivery of molecules into cells, thereby expanding the technology's applications beyond extraction to advanced bioprocessing.

## Author Contributions

**Byron Perez**: conceptualization, investigation, writing – original draft, visualization. **Julia Baumgartner**: conceptualization, investigation, writing – review and editing. **Daniel Macken**: conceptualization, investigation, writing – review and editing. **Iris Haberkorn**: conceptualization, supervision, writing – review and editing, funding acquisition. **Alexander Mathys**: conceptualization, writing – review and editing, supervision, funding acquisition.

## Funding

We thank the Bezos Earth Fund for their support to the Bezos Centre for Sustainable Protein at NUS. This work was supported by the National Research Foundation Singapore, under its Campus for Research Excellence and Technological Enterprise (CREATE) program (Award No. NRF2020‐THE003‐0004, 2020). J. Baumgartner was supported by the Swiss National Science Foundation, Project No. 198750. D. Macken was supported by Uniscientia Stiftung via the ETH Zurich Foundation project number 2023‐HS‐430.

## Conflicts of Interest

The authors declare no conflicts of interest.

## Data Availability

The images and cultivation information of Figure 4 can be accessed through the ETH research collection: https://doi.org/10.3929/ethz‐c‐000791013. No other new research data were generated for this manuscript.
